# Why ruminating ungulates chew sloppily: Biomechanics discern a phylogenetic pattern

**DOI:** 10.1371/journal.pone.0214510

**Published:** 2019-04-17

**Authors:** Zupeng Zhou, Daniela E. Winkler, Josep Fortuny, Thomas M. Kaiser, Jordi Marcé-Nogué

**Affiliations:** 1 School of Mechanical and Electrical Engineering, Guilin University of Electronic Technology, Guilin, China; 2 Johannes Gutenberg University Mainz, Institute of Geosciences, Mainz, Germany; 3 Institut Català de Paleontologia Miquel Crusafont, Universitat Autònoma de Barcelona, Edifici ICTA-ICP, c/ Columnes s/n, Barcelona, Spain; 4 Centre de Recherches en Paléontologie de Paris, Muséum National d'Histoire Naturelle, Bâtiment de Paléontologie, Paris, France; 5 Centrum für Naturkunde, University of Hamburg, Hamburg, Germany; Liverpool John Moores University, UNITED KINGDOM

## Abstract

There is considerable debate regarding whether mandibular morphology in ungulates primarily reflects phylogenetic affinities or adaptation to specific diet. In an effort to help resolve this debate, we use three-dimensional finite element analysis (FEA) to assess the biomechanical performance of mandibles in eleven ungulate taxa with well-established but distinct dietary preferences. We found notable differences in the magnitude and the distribution of von Mises stress between Artiodactyla and Perissodactyla, with the latter displaying lower overall stress values. Additionally, within the order Artiodactyla the suborders Ruminantia and Tylopoda showed further distinctive stress patterns. Our data suggest that a strong phylogenetic signal can be detected in biomechanical performance of the ungulate mandible. In general, Perissodactyla have stiffer mandibles than Artiodactyla. This difference is more evident between Perissodactyla and ruminant species. Perissodactyla likely rely more heavily on thoroughly chewing their food upon initial ingestion, which demands higher bite forces and greater stress resistance, while ruminants shift comminution to a later state (rumination) where less mechanical effort is required by the jaw to obtain sufficient disintegration. We therefore suggest that ruminants can afford to chew sloppily regardless of ingesta, while hindgut fermenters cannot. Additionally, our data support a secondary degree of adaptation towards specific diet. We find that mandibular morphologies reflect the masticatory demands of specific ingesta within the orders Artiodactyla and Perissodactyla. Of particular note, stress patterns in the white rhinoceros (*C*. *simum*) look more like those of a general grazer than like other rhinoceros’ taxa. Similarly, the camelids (Tylopoda) appear to occupy an intermediate position in the stress patterns, which reflects the more ancestral ruminating system of the Tylopoda.

## Introduction

The main function of the mammalian mandible is to transmit the forces generated by masticatory muscles to the teeth for food processing [1]. The shape of the mandible has been proposed to reflect the frequency and magnitude of muscle activation, bite force, and the properties of the food ingested, while ensuring structural integrity under all loads without dissipation or failure [2]. In general, the greater the force required to fracture ingesta, and the more repeatedly such forces need to be produced, the stiffer the mandible has to be to maintain its structural integrity [3]. For example, it has been demonstrated that among primates, hard-food consumers have stiffer mandibles compared to soft-food consumers [4].

The relationship between bite force, size and shape of the mandible in relation to ingesta has been studied previously in several mammalian clades including Artiodactyla [5–7], Chiroptera [8–12], Primates [13–15] and Carnivora [16–19]. Due to the close interaction between the mammalian feeding mechanism and diet, the biomechanical study of extant species can illuminate ecomorphological adaptations to provide accurate predictions of extant species and potentially acquire valuable tools for the reconstruction of oral behaviour in extinct taxa [20–22].

In ungulates, the hypsodonty index (HI), among other morphological trait, and, to a lesser extent, the moment arms of jaw muscles have been used to infer dietary traits in extinct species [7,23]. The hypsodonty index relates to ingesta abrasiveness and clearly increase in grazers due to higher dietary abrasive component [24]. For example, Varela and Fariña [7] demonstrated a relationship between the HI, masseter moment arm, and the amount of grass in the habitat. It is further well established that grazers tend to have more robust mandibles and larger masseters compared to browsing species, because they have to comminute the relatively tough, highly fibrous ingesta more thoroughly [25]. Mendoza and Palmqvist [26] demonstrated that the length of the mandible and the HI are higher in ungulates from open (hence dustier) habitats, whereas a wider muzzle is common in bulk, non-selective grass feeders. These observations suggest that mandible shape variation between species can be linked to a different mode of feeding especially when comparing specialized browsers with grazers. However, mandible movement types are also constrained by developmental processes so that interspecific variation might reveal strong phylogenetic signal in morphological design and performance [6,17,27–29]. This phylogenetic signal is found to be stronger in Perissodactyla than Artiodactyla [30]. The degree to which mandibular morphology is influenced by diet, reflects phylogenetic affinities or both is thus still debated. Biomechanical data, which better characterizes ungulate mandibular diversity, may shed more light on this relationship and help to disentangle contradictory interpretations.

Finite element analysis (FEA) is an ideal technique to provide a comparative perspective on mandibular biomechanics. Recently, FEA was used to assess stress during mastication in the mandibles of different vertebrate taxa using 3D models [31–34] and plane models [35–38]. In a comparative context, FEA studies have employed a variety of approaches including qualitative and quantitative approaches. Qualitative approaches include stress distribution plots, while quantitative methods involve examining stress at particular points, comparing model means and combining FEA with geometric morphometrics to compare model distortion [39]. Both, quantifying stress data at particular points [38,40–46] as well as taking into account the strength of the whole model by computing von Mises stress averages, have proven useful in ecomorphological analyses [37,47–54]. FEA outputs have been used to explore functional morphology, ecomorphology and macroevolution, by applying standard statistical methods [35,55] or geometric morphometrics to analyse deformations after load application [56].

In this study, we employed “weight-meshed values” and “quasi-ideal meshes” to compute the values of stress recently proposed by Marcé-Nogué et al. [4,34,57]. This method is more robust compared to other comparative approaches because it considers the effects caused by the different size and homogeneity of the mesh elements in the FEA model. We also employed the new intervals’ method to analyse data from FEA models in a comparative multivariate framework [58,59]. This method allows for considerably more effective comparison of FEA models, and consequently more precise distinction between feeding strategies. Both, the quantification of average values of stress [57] and the use of the intervals’ method [58], have been previously employed to discriminate dietary preferences in the study of the biomechanical traits of armadillo mandibles by separating the results of stress as a function of diet.

In the present study we compared 3D finite element models of mandibles of Artiodactyla (*Alcelaphus buselaphus*, *Aepyceros melampus*, *Camelus dromedarius Giraffa Camelopardalis*, *Lama glama* and *Litocranius walleri*) and Perissodactyla (*Ceratotherium simum*, *Diceros bicornis*, *Dicerorhinus sumatrensis*, *Equus quagga* and *Tapirus terrestris)* selected because they cover different examples of proportions of grass in their diets [60,61]. We analysed lateral and orthal biting action and explore the degree to which morphology reflects adaptation towards diet or underlying phylogenetic relationships.

## Materials and methods

### Specimens

A total of 11 extant ungulate taxa were analysed. All specimens examined are housed either at the Centrum für Naturkunde in Hamburg (ZMH), Germany, the Museum für Naturkunde Berlin (ZMB), Germany, the Museum Victoria in Melbourne (NMV), Australia or in the Texas Memorial Museum (TMM), USA. All the specimens are adult individuals with no pathology. See Table 1 for further details. Species were classified according to dietary trait as follows [60,62]: GGR: general grazer, GBR: general browser, OMF: open-habitat mixed-feeder and HBR: high-level browser (Table 1).

**Table 1:** List of ungulate species investigated.

SPECIES	ID	ORDER	DIET	AGE/SEX	STATUS
*Alcelaphus buselaphus*	ZMH-S-7487	Artiodactyla	GGR	AF	Captive
*Aepyceros melampus*	ZMH-S-10162	Artiodactyla	OMF	YAM	Captive
*Camelus dromedarius*	NMV R 5444	Artiodactyla	OMF	AM	Captive
*Ceratotherium simum*	ZMH-S-2552	Perissodactyla	GGR	SAF	Wild
*Diceros bicornis*	ZMH-S-9379	Perissodactyla	GBR	Adult-U	Wild
*Dicerorhinus sumatrensis*	ZMH-S-8273	Perissodactyla	GBR	Adult-U	Wild
*Equus quagga*	ZMB-Mam-70335	Perissodactyla	GGR	U	Wild
*Giraffa camelopardalis*	NMV C 32820	Artiodactyla	HBR	Adult-U	Captive
*Lama glama*	TMM M-2052	Artiodactyla	OMF	Female	U
*Litocranius walleri*	ZMB-Mam-39663	Artiodactyla	HBR	U	Wild
*Tapirus terrestris*	TMM M-16	Perissodactyla	GBR	Subadult-U	U

Museum Acronyms: ZMH = Centrum für Naturkunde, Hamburg, Germany, ZMB = Museum für Naturkunde, Berlin, Germany, TMM = Texas Memorial Museum, Austin, USA and NMV = Museum Victoria, Melbourne, Australia); Diet classification after Mendoza et al. [60] GGR: general grazer, GBR: general browser, OMF: open-habitat mixed-feeder and HBR: high-level browser. YAM: young adult male, SAF: Subadult female, AF: Adult female, AM: Adult male, U: Unknown.

### Geometric reconstruction

High resolution CT scans of *Alcelaphus buselaphus*, *Aepyceros melampus*, *Equus quagga* and *Litocranius walleri* were obtained at Steinmann-Institut für Geologie, Mineralogie und Paläontologie (Universität Bonn, Germany) using a CT scanner (Philips Brilliance 64). Specimen dimensions ranged between approximately 20–40 cm in length resulting in voxel sizes between 0.456 mm and 0.793 mm. Scans of *Diceros bicornis* and *Ceratotherium simum* were performed at Universitätsklinikum Hamburg Eppendorf (UKE), Germany, on a medical Philips Brilliance 64 CT scanner (voxel size 0.977 mm and 0.651 mm respectively). *Lama glama* (voxel size 0.1768 mm) and *Tapirus terrestris* (voxel size 0.5918 mm) were scanned on a medical CT at The University of Texas High-Resolution X-ray CT Facility (UTCT) (see www.digimorph.org for further details). *Giraffa camelopardalis* (voxel size of 0.976 mm) and *Camelus dromedarius* (voxel size 0.804 mm) were scanned in a medical CT (Siemens Sensation 64 scanner) at St. Vincent's Hospital, Melbourne, Australia, and *Dicerorhinus sumatrensis* was scanned in a medical CT at Yxlon facilities in Hamburg, Germany using a Y.CT Modular (YXLON International GmbH) equipped with a Y.TU450-D11 x-ray tube (voxel size of 0.23 mm).

CT datasets were imported to the software Avizo 7.0 (FEI-VSG Company). The scans were manually segmented and a surface model was reconstructed with multiple materials. Molar teeth (M1, M2 and M3) were reconstructed as a separate material from the mandibular bone, as they bear the highest functional load in comminution [63]. Molars were then included in the models as auxiliary geometry to apply the force in the bite point. During this step, irregularities in the surface resulting from model generation during scanning were repaired using Rhinoceros 5.0 software (McNeel & associates) refinement and smoothing tools. The segmented models were converted to a CAD models following Marcé-Nogué et al. [64]. A CAD model is a vector-graphic standard for three-dimensional design used in mechanical design and engineering which does not involve any tetrahedralization of the geometry such as stereolithographic formats. The CAD-step allowed us to create the mesh of finite elements in ANSYS FEA Package. All models were standardized and oriented in anatomical position with maxilla and mandible articulated to facilitate comparison.

### Model properties

A structural static analysis was performed to evaluate the biomechanical performance of the mandible during biting using the finite element package ANSYS 17.1 (Ansys, Canonsburg, USA) and a Dell Precision Workstation T7910 with 256GB. Our primary interest was the comparison of von Mises stress distribution throughout the mandible under loading conditions defined by masticatory movement. The von Mises criterion is the most accurate value for predicting fracture location when isotropic material properties are applied to cortical bone [65]. For this study, elastic, linear and homogeneous material properties were assumed for bone tissue. Although the specific mechanical properties of bone for the investigated individuals are unknown, Gil et al. [66] demonstrated that the use of general values for bone does not alter results to an extent that a relative comparison between models is inappropriate. Similarly, Strait et al. [67] found that models with heterogeneous mechanical properties of bone closely matched models assuming homogenous properties. Therefore, values from bovine haversian bone: E [Young´s modulus] = 10 GPa and v [Poisson ratio] = 0.4)[68] and for tooth enamel E = 50.14 GPa and v = 0.3 [69] were used and homogenous mechanical properties were assumed for all bones in this study. The high stiffness of the molars compared with the bone makes the molars act as a rigid body that helps the transmission of the forces to the mandible. The mandibles were meshed using the ANSYS mesh module with an adaptive mesh of hexahedral elements [70]. The mesh of each model was approximately between 0.8–1.5 million elements.

### Boundary and loading conditions

**Fig 1:**
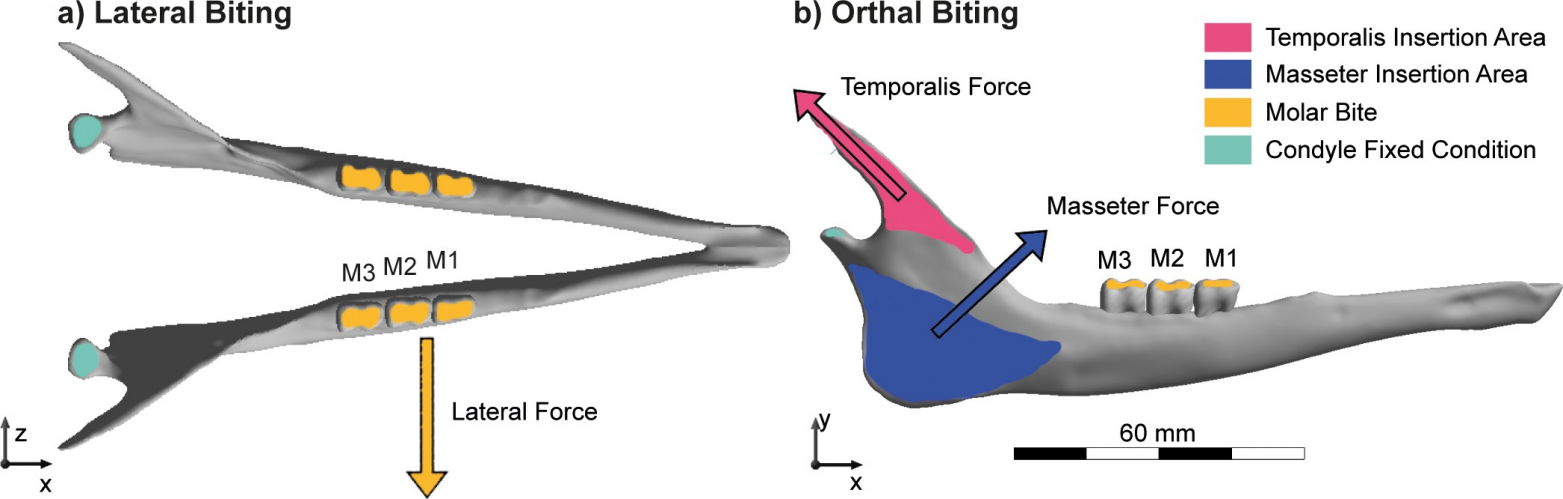
**Free-body diagram** of the biomechanical problem with boundary conditions, muscular forces, area of insertion, and bite position for each molar (M1, M2, M3) in *L*. *walleri* for a) lateral and b) orthal biting configurations.

Two bite configurations: lateral and orthal, were simulated based on configurations described by Fortuny et al. [71] and Fletcher et al. [36], respectively (Fig 1). Bite positions were placed on the first, the second, and the third molar separately, generating six cases per individual. The lateral bite configuration was defined using extrinsic loading and the application of force in the z-direction in the plane of the molar row, resulting in a lateral chewing motion [72]. It needs to be noted that postcanine orthal biting is a less significant component of masticatory movements in herbivorous ungulates where lateral power stroke component predominate in comminution. Orthal biting action however would apply on ingestion when using incisors. Previous works on ungulates [36] tested digestive physiology versus mandible stiffness in orthal bite but did not investigate lateral movements, although lateral occlusal movements are the basic chewing strokes in ungulates.

For the orthal bite configuration, boundary and loading conditions were defined using intrinsic loading and including only the masseter and temporalis muscles. The insertion areas of both muscles were defined in the model in order to apply the muscle forces during biting. The direction of these forces was defined by the line that joins the centroid of the area of origin on the cranium with its attachment point on the mandible. Because the force that a muscle can produce depends on its active cross section, the size of the attachment area for each muscle was used to define the application of muscle tension at the point of attachment. However, as the interest of the study is a comparative one, instead of calculating absolute values for forces, we used these areas to quantify the proportional amount of force developed by each muscle assuming a muscular pressure of 0.3 MPa [73]. To simulate a bite, a fixed displacement boundary condition in y-direction was applied in the bite locations to model the moment at which cranium and mandible contact the food.

The six bite cases simulated were: 1. Lateral first molar biting, 2. Lateral second molar biting, 3. Lateral third molar biting, 4. Orthal first molar biting 5. Orthal second molar biting and, 6. Orthal third molar biting.

In all cases, restrictions in displacement were applied at the occipital condyles to fix the mandible in the x and y-direction in relation to the vertebral column (Fig 1).

### Muscle force: Scaling the models

To compare the performances of structures that differ in shape and size, the values of muscular contraction transmitted to bone were calculated according to the methodology developed by Marcé-Nogué et al. [74] and adjusted for 3D models by Fortuny et al. [75]. This methodology uses allometric principles to relate mandibular volume for each specimen with the muscular forces applied by a 2/3 power relationship and agrees with the allometric proportions of the species [41,76,77].

To obtain calibration values for force (lateral bite configuration) and muscular pressure (orthal bite configuration), *Litocranius walleri* was employed as a reference model, with a value of 0.3 MPa assumed for muscular contraction pressure [73]. Models for each of the remaining species were scaled against *L*. *walleri* according to their respective difference in mandibular volume.

According to the equation proposed by Fortuny et al. [75] muscular pressure (P) is defined as force divided by the area of muscular insertion (MI) for each muscle. The muscular pressure of both models A and B are related to variation in the volume (V) of the mandible (Eq 1 and Table 2).

$$P_{A}=\frac{{MI}_{B}}{{MI}_{A}}{\left(\sqrt[{3}]{\frac{V_{A}}{V_{B}}}\right)}^{2}P_{B}$$ (Eq 1)

**Table 2:** Geometric properties and muscular pressure. Volume of the mandible [mm^3^], masseter and temporalis area [mm^2^] and muscular pressure [MPa] applied in the FEA model for each species.

Model	Volume of the mandible [mm3]	Lateral Bite Cases 1,2 and 3	Orthal Bite Cases 4, 5 and 6			
		Lateral force [N]	Masseter Area [mm^2^]	Temporalis Area [mm^2^]	Masseter Pressure [MPa]	Temporalis Pressure [MPa]
*A*. *buselaphus*	372240	204.91	5334	1932	0.3226	0.3333
*A*. *melampus*	114826	93.55	1551	510	0.5063	0.5768
*C*. *dromedarius*	967170	387.28	8366	1542	0.3887	0.7894
*C*. *simum*	5275100	1199.98	26769	3137	0.3764	1.2026
*D*. *bicornis*	3266000	871.70	12406	1972	0.5900	1.3897
*D*. *sumatrensis*	1531700	526.18	10254	1169	0.4309	1.4146
*E*. *quagga*	802150	341.87	12671	1059	0.2265	1.0151
*G*. *camelopardalis*	1133000	430.37	10824	2763	0.3339	0.4896
*L*. *glama*	198380	134.69	3104	959	0.3643	0.4416
*L*. *walleri*	20852	30.00	841	314	0.3000	0.3000
*T*. *terrestris*	823910	348.02	9414	2883	0.3104	0.3795

### Average values and quasi-ideal mesh

The distribution of von Mises stress is useful in biomechanics because von Mises stress patterns indicate relative strength. For instance, specimens that are characterized by higher values of stress are considered less resistant to the effect of the forces. In this study, we used von Mises stress maps of the mandibles, the average values of von Mises stress and box-plots of the stress distribution in each taxon to investigate mandibular strength. Average values of von Mises stress were evaluated following the method proposed by Farke [47], who recommends plotting stress distributions as quantitative data. However, the use of box-plots for stress distributions and the statistics derived from them (e.g. percentiles or whiskers) requires the use of a quasi-ideal mesh (QIM) which necessitates corrections for the non-uniformity of the mesh. For non-uniform meshes (where different elements have different sizes), new statistics, which take into account non-uniformity such as the mesh-weighted arithmetic mean (MWAM) [57] have been proposed. The phylogenetic signal was estimated for the biomechanical data using a mathematical generalization of the K-statistic appropriate for multivariate data (i.e. Kmult) [78].

For the mandibles, the MWAM, and the stress quartile values given in the boxplots, the percentage error of the arithmetic mean (PEofAM) and the percentage error of the median (PEofM) (described in Marcé-Nogué et al. [57]), as well as the values needed to calculate both percentage errors, can be found in Supplementary information (S1 and S2 Tables) All analyses were performed in Matlab R2017a (Mathworks, Massachusetts, USA).

### Cluster analysis

Cluster analyses (Ward's method for agglomerative-hierarchical analysis) were performed using the Von Mises stress distribution (MWAM and percentiles) data. Two different cluster analyses were performed: one using the average MWAM values and the second using the percentiles of the six biting cases. Euclidean distances were used as the similarity index [79], cophenetic correlation coefficient to show the stability of the cluster [80] and a dendogram was generated using the results to determine affinities between taxa in the biomechanical data. The data was scaled and centred to improve numerical stability. All analyses were performed in R v. 3.4.4. [81].

### Intervals’ method

A new variable hereafter referred to as the “vector for stress intervals” representing a different interval of stress values was created following the interval method [58]. The interval method involves a new organization of the stress values of the models subdividing stress values obtained into defined intervals, with each interval representing an subarea (as a percentage) of the original model and reflecting a specific range of stress.

The interval method described by Marcé-Nogué et al. [58] requires the definition of a fixed upper threshold FT_upper_ = 15 MPa for the three lateral bite cases and a fixed upper threshold FT_upper_ = 25 MPa for the three orthal bite cases as well as a specific number of intervals (N = 100). The number of intervals for our analyses were determined using the convergence procedure described by Marcé-Nogué et al.[58] (S1, S2, S3, S4, S5 and S6 Figs and S3 Table of the supplementary information). Although fewer intervals could have been be used for the orthal cases, we decided to standardize all the analysis to N = 100, which is suitable for both orthal and lateral cases. The values of each interval when N = 100 for each specimen and for each biting case can be found in the supplementary information (S1 Document).

Subsequently, these new vectors for stress intervals were analysed using multivariate methods such as principal component analysis (PCA) and plotted in a biplot. All analyses were performed using Matlab (Mathworks, Massachusetts, USA) and R v. 3.4.4. [81]. The phylogenetic signal was also estimated for the intervals’ method data using a mathematical generalization of the K-statistic appropriate for multivariate data.

## Results

### Von mises stresses

**Fig 2:**
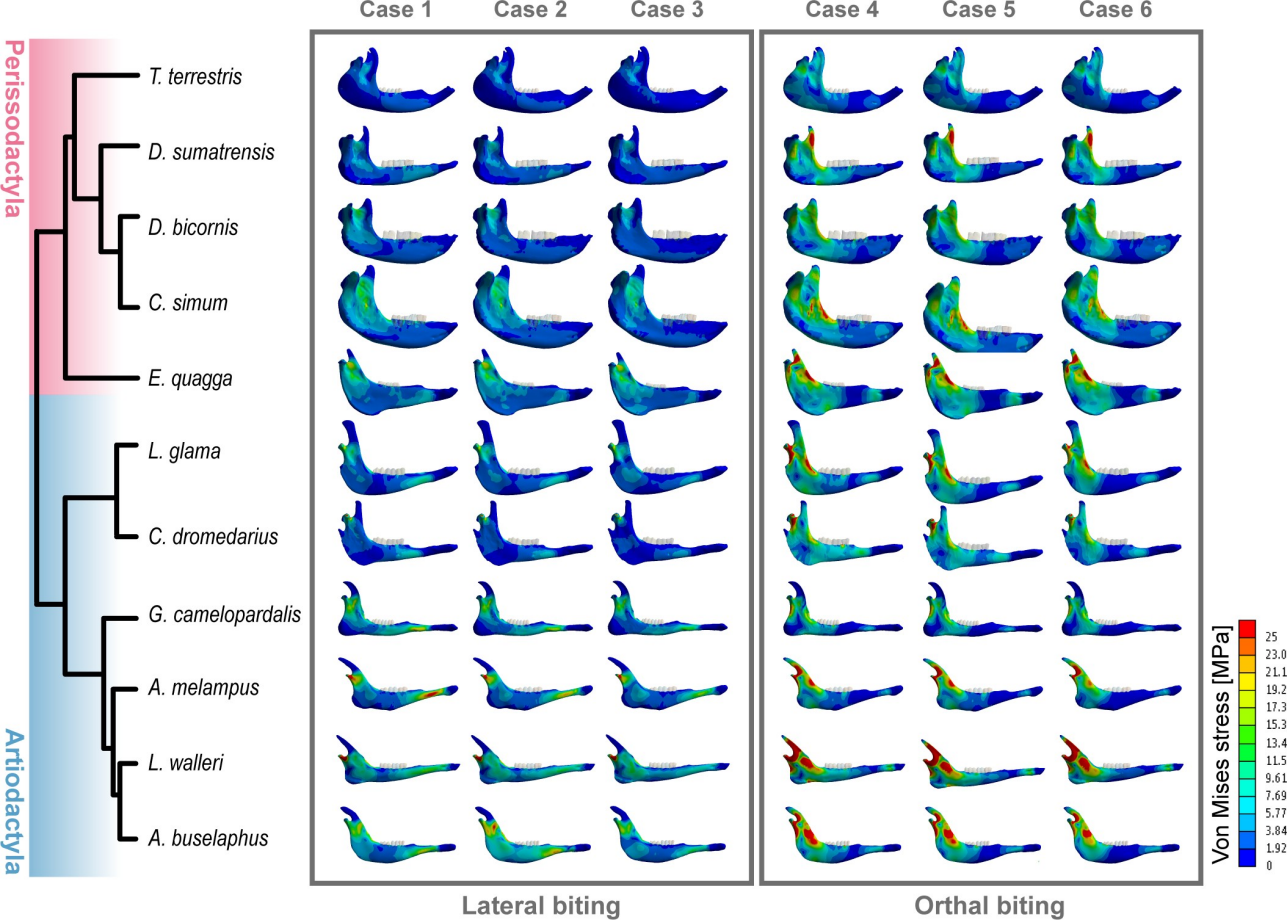
Phylogenetic tree and modelled von mises stress distribution in the mandible and the molars for each taxon for each bite configuration and bite position. Lateral cases 1) in M1 2) in M2 and 3) in M3. Orthal cases 4) in M1 5). In M2 and 6) in M3. Phylogenetic tree from http://10ktrees.nunn-lab.org/.

A general pattern distinguishes the orders Artiodactyla and Perissodactyla. Artiodactyla mandibles are characterized by more areas of high stress in the ramus whereas Perissodactyla exhibit lower stress values, particularly across large areas of the corpus (Fig 2). The three lateral bite cases 1, 2 and 3 showed high stresses in the ramus with the highest stress occurring in the condyle (Fig 2). The orthal bite cases 4, 5 and 6 exhibited an important area of lower stress in the region between the bite position (molars) and the mandibular symphysis. All mandibles showed a high level of stress at the mandibular notch as well as from the condyle through the ramus in a descending gradient.

**Fig 3:**
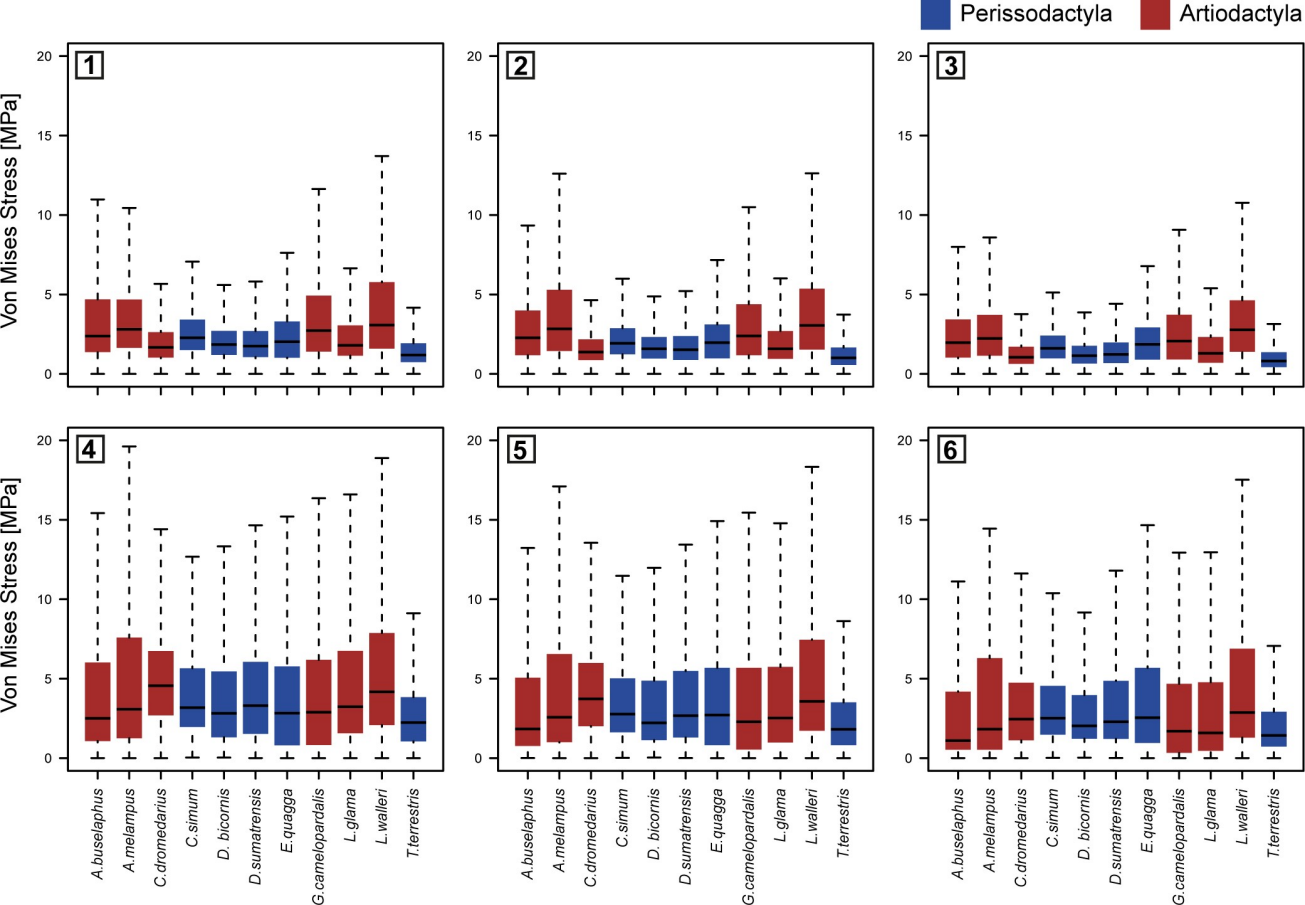
Box-plots of modelled von mises stress distributions in the mandible by taxon for each bite configuration and bite position. Lateral cases 1) in M1 2) in M2 and 3) in M3. Orthal cases 4) in M1 5). In molar M2 and 6) in M3. Quasi-Ideal Mesh (QIM) was assumed. Upper and lower whiskers indicate 90% thresholds of the von Mises stress distributions.

Quantitative methods were utilized to better understand the intensity of the stresses in the mandibles. Boxplots of stress distributions (assuming Quasi-Ideal Mesh (QIM)) are depicted by taxon for both bite configurations in Fig 3. Average MWAM values and the quartiles values for the boxplots are listed in S1 Table (lateral cases) and S2 Table (orthal cases) of the supplementary information: M25 corresponds to the 25^th^ percentile, M50 to the 50^th^ percentile, M75 to the 75^th^ percentile and M95 to the 95^th^ percentile.

An important caveat to the FEA method is that singular and unusually high stress values can appear where the boundary conditions are set. These numerical singularities are an artefact of the applied mathematical approach, inflated by the constraints imposed on the model [70]. In these areas, stresses have the tendency to increase in value towards infinity. However, these singularities are not related to any biological process and thus should be excluded from qualitative analyses of stress. The suggestions of Walmsley et al. [82], who recommend using the 95^th^ percentile (M95) as the peak stress value in analyses in order to avoid these artificially high values are followed here.

**Fig 4:**
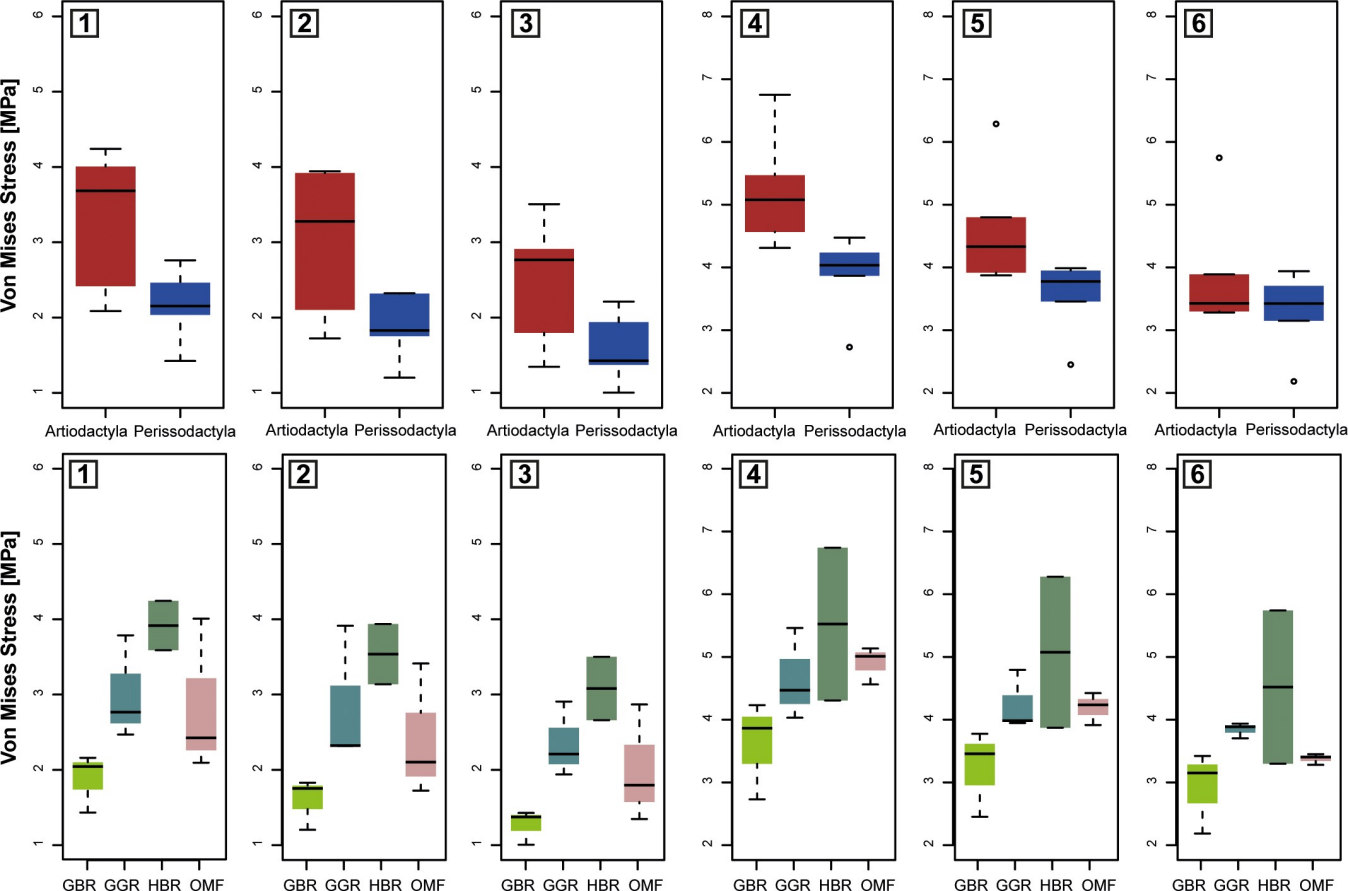
Boxplots of averaged stress MWAM values of all species grouped by order (Artiodactyla and Perissodactyla) and by dietary categories (GGR: General grazer, GBR: General browser, OMF: Open-habitat mixed-feeder and HBR: High-level browser). Lateral cases 1) in M1 2) in M2 and 3) in M3. Orthal cases 4) in M1 5) in M2 and 6) in M3. Central line is the median, whiskers represent the range.

**Fig 5:**
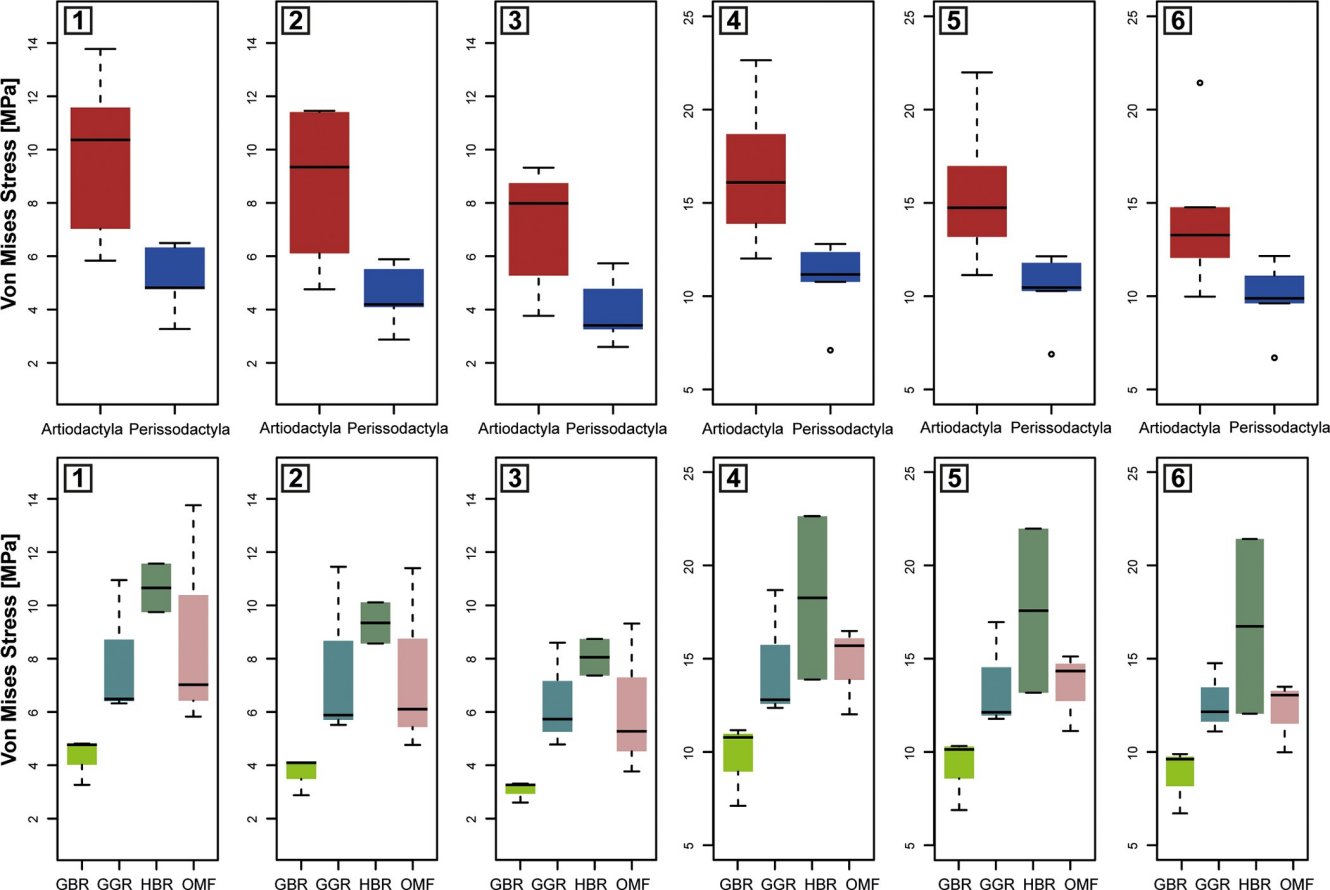
Boxplots of the peak stress values (assuming the 95^th^ percentile as the peak stress value) for all species grouped by order (Artiodactyla and Perissodactyla) and by dietary categories (GGR: General grazer, GBR: General browser, OMF: Open-habitat mixed-feeder and HBR: High-level browser). Lateral cases 1) in M1 2) in M2 and 3) in M3. Orthal cases 4) in M1 5) in M2 and 6) in M3. Central line is the median, whiskers represent the range.

Comparison of averaged stress values (MWAM) (Fig 4) and peak stress values (Fig 5), reveal differences between Artiodactyla and Perissodactyla in both bite configurations and suggest that Perissodactyla have stiffer mandibles than Artiodactyla.

However, both averaged stress values (MWAM) and peak stress values (M95) also appear to capture differences in dietary traits (Figs 4 and 5). For instance, the mandibles of *D*. *bicornis*, *D*. *sumatrensis* and *T*. *terrestris*, all categorized as general browsers in this comparison, are characterized by lower stress values than general grazers and mixed feeders (intermediate stress values) and high-level browsers (highest stress values), suggesting that these taxa have stiffer mandibles despite their browsing activity).

**Fig 6:**
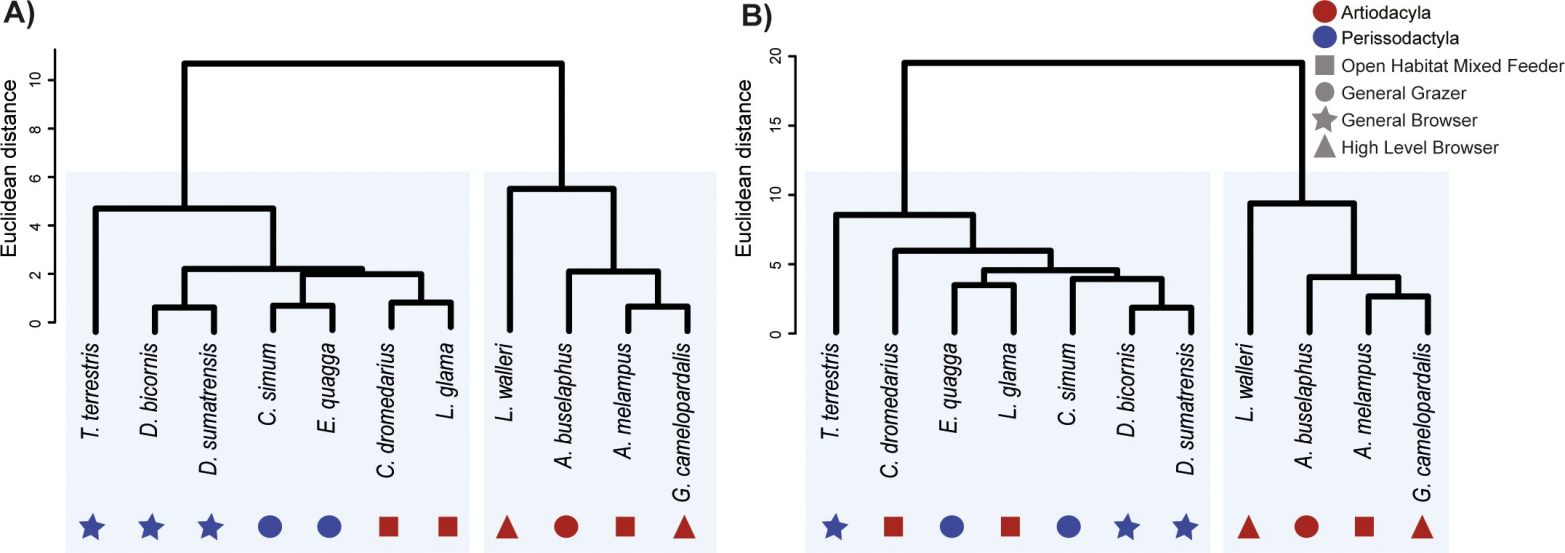
**Hierarchical clustering analysis** using Ward's method for a) averaged stress values (MWAM) values and b) percentiles in all the six biting cases.

Cluster analyses show that species group according to their taxonomic group if averaged MWAM are used (Fig 6A) although the camel and the lama both cluster with perissodactyls. The same result indicating affinities between perissodactyls (Perissodactyla) and camelids (Tylopoda) is obtained if percentiles are used (Fig 6B). Cophenetic correlation coefficient for both clusters was obtained (R_MWAM_ = 0.64 and R_percentiles_ = 0.66).

**Fig 7:**
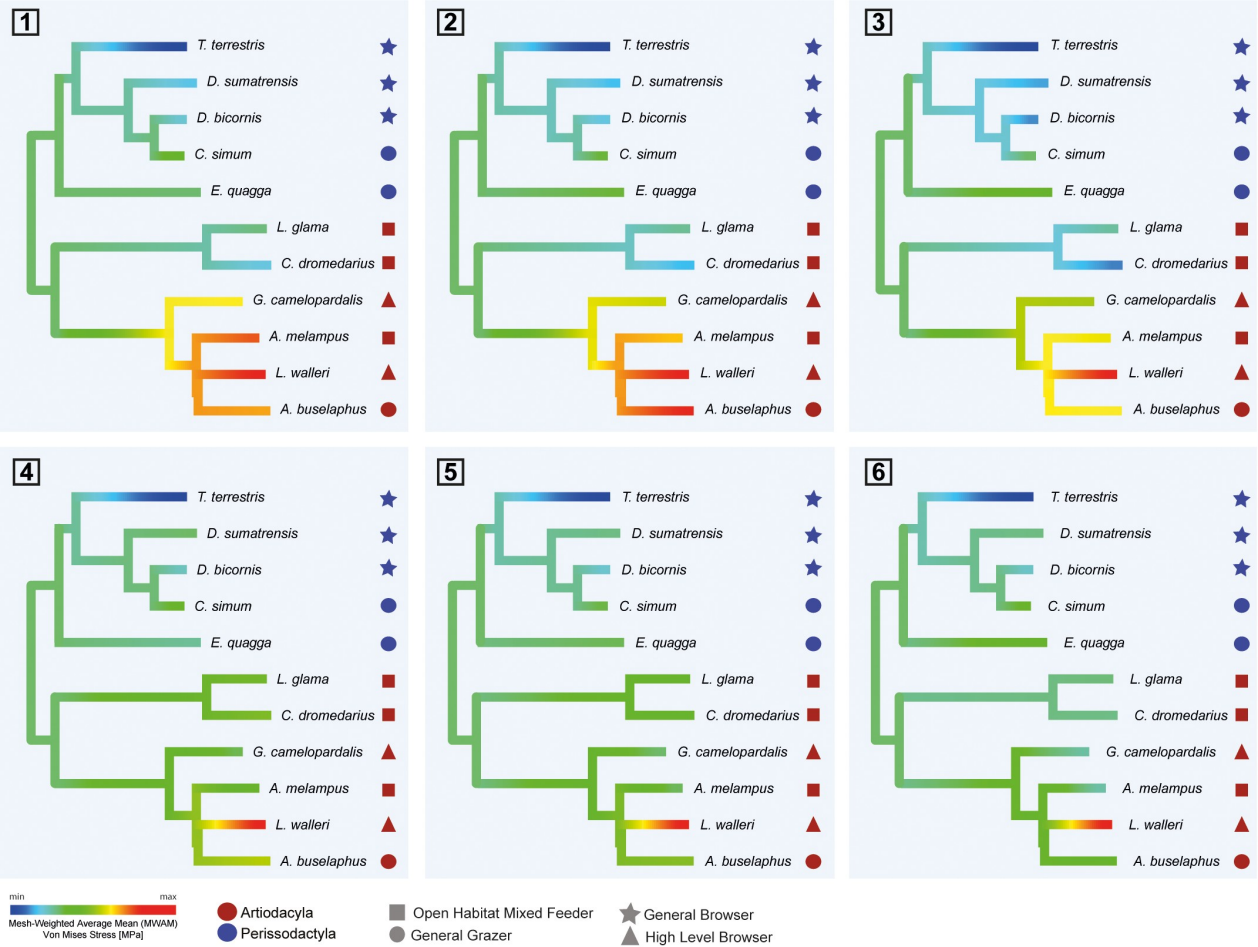
Stress values MWAM on the phylogeny for the six biting cases: Lateral cases 1) in M1 2) in M2 and 3) in M3. Orthal cases 4) in M1 5) in M2 and 6) in M3. The colour ranges from red representing higher average stress values, to blue, representing lower stress values.

Fig 7 shows the results of the ancestral state reconstruction for the averaged MWAM stresses in the six bite scenarios. These values were mapped on the phylogeny using a maximum-likelihood ancestral character estimation method based on a Brownian motion model of evolution. The figure depicts the same results of cluster analysis where species presented similar results according to their taxonomic group but camelids with the order of magnitude of perissodactyls. Finally, Significant phylogenetic signal was found for MWAM data of the six bite cases (Kmult = 1.06; p-value = 7e-4; 9999 permutations) and percentile data of stress (Kmult = 1.24; p-value = 3e-4; 9999 permutations).

### Intervals’ method

**Fig 8:**
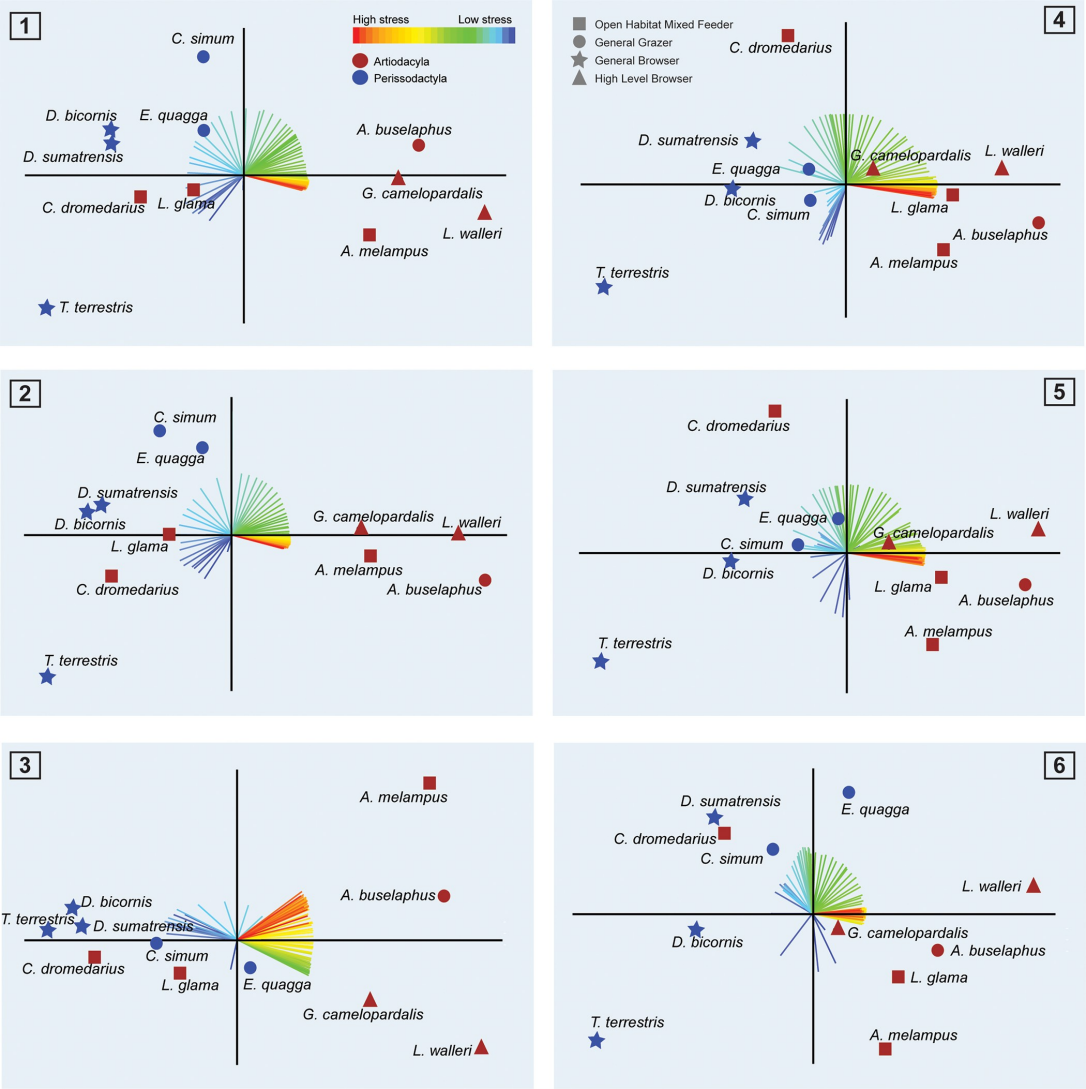
Biplot of PCAs based on the correlation matrix for the six biting cases: Lateral cases 1) in M1 2) in M2 and 3) in M3. Orthal cases 4) in M1 5) in M2 and 6) in M3. The loadings for each variable are coloured according stress intensity. Red colours indicate high stress, blue indicate low stress. X-axis: PC1. Y-axis: PC2.

Biplots of stress space for the first two principal components of the PCA indicate that multiple variables contribute importantly to the PCs analysed (Fig 8). Variance of the first two components explain more than 80% of the variance in lateral and orthal cases (see S4 Table for the percent of the variance explained by the first ten components in all six cases). Perissodactyla are characterized by negative PC1 values which represents overall lower stress. *Tapirus terrestris* exhibiting also negative scores in PC2, because it has large areas of very low stress whereas other Perissodactyla with fewer and smaller areas of low stress present positive PC2 values. Artiodactyla exhibit primarily positive scores in PC1 because their mandibles are characterized by larger areas of high and moderately high stress. A notable exception to this pattern are the Tylopoda (*C*. *dromedarius* and *L*. *glama)* which, for the lateral bite case, present negative PC1 scores (more areas of intermediate-low stress) close to some of the Perissodactyla. For the orthal bite case, *L*. *glama* plots near the Ruminantia (*A*. *buselaphus*, *A*. *melampus*, *G*. *Camelopardalis* and *L*. *walleri*) and *C*. *dromedaries*, with large areas of intermediate values of stress, plots between the Ruminantia and the Perissodactyla. Finally, Significant phylogenetic signal was found for MWAM data of the six bite cases (Kmult = 0.78; p-value = 0.0012; 9999 permutations). The PC plots do not clearly differentiate dietary traits.

## Discussion: Diet-related stress patterns versus phylogenetic patterns

In this study, we generated FEA models to test whether the biomechanical performance of the ungulate mandible reflects more feeding adaptations or historical contingencies. Our data include eleven species of ungulate with distinct, well-established dietary traits but belonging to two different clades, Perissodactyla and Artiodactyla.

Our results reveal clear differences between Perissodactyla and Artiodactyla (Figs 2, 4 and 5). The mandibles of these taxa differ in biomechanical performance during orthal and lateral biting and in their mandibular movements during chewing and suggesting that Perissodactyla have stiffer mandibles than Artiodactyla. However, differences are also evident between taxa with distinct dietary traits. For instance, von Mises stress distributions (Figs 4 and 5), indicate that general browsers have comparatively stiffer mandibles than general grazers and mixed feeders while highly specialized browsers possess the most fragile mandibles.

These patterns are not necessarily reflective of dietary adaptations only, but may be biased by phylogeny, particularly when closely related taxa have undergone an adaptive radiation into different dietary niches. The latter scenario is suggested by the significant differences observed in this study between members of Artiodactyla (Ruminantia and Tylopoda) and Perissodactyla in both average and peak von Mises stress values which seem independent of dietary trait assignment (S1 and S2 Tables of the Supplementary information). Cluster analysis of stress values (Fig 6A and 6B) and the intervals’ method (Fig 8) shed further light on this issue. Cluster analysis of averaged stress values (MWAM) and the percentiles both resulted in two distinct clusters: one including Tylopoda and Perissodactyla and the other including the Ruminantia (Fig 6). Similarly, intervals method reveal that Perissodactyla and Ruminantia are different in mandibular stress distribution because they are placed in a different area of the PC plots.

Apparent phylogenetic differences are more evident between Perissodactyla and ruminant species. The strong phylogenetic signal supported by Kmult values in mandibular stress levels we observe between ruminants and Perissodactyla is likely related to major divergence in the digestive physiology of the two groups. Perissodactyla are hindgut fermenters and have shorter gut passage times compared to Ruminantia and Tylopoda of similar body mass and feeding trait. Hindgut fermenters thus need to compensate for this with higher food intake rates [83–85]. Fletcher et al. [36] suggest that extant hindgut fermenters have more robust mandibles than ruminants—regardless of their dietary traits—as a result of their higher food intake rates. Conversely, the more gracile mandibles of ruminants likely result from their ability to re-masticate their food several times after initial ingestion by regurgitating and chewing their cud. Rumination results in re-hydration and softening of food as well as internal ‘washing’ of ingesta in the rumen [86], which removes hard, exogenous grid from food surfaces. Moreover, splitting mastication into several cycles may reduce the overall mechanical demand of chewing by permitting less thorough mastication and necessitating lower bite forces upon initial ingestion. All of these variables may contribute to a release on masticatory demands. Although Fletcher et al. [36] investigated orthal bite configurations only; our FEA results are nonetheless consistent with their data in both orthal and lateral bite configurations. In general, it would appear that ruminants can afford to chew sloppily upon initial digestion, while hindgut fermenters cannot, necessitating stronger mandibles with higher stress resistance in the latter group.

The separation of Ruminantia from Tylopoda, both of which belong to the order Cetartiodactyla, suggest a non-phylogenetic influence on the evolution of even-toed ungulate mandibular morphology. For example, *C*. *dromedarius* and *L*. *glama* clearly differ from ruminants in their stress distribution. In fact, both cluster statistics and intervals method PC plots indicate affinities between perissodactyls (Perissodactyla) and camelids (Tylopoda). Interestingly, cluster analyses using averaged stress values (MWAM) suggest that inside the rhinocerontids these affinities in mandibular morphologies could reflect dietary preferences (Fig 6).

In the PC plots of lateral biting configurations, both Tylopoda taxa have intermediate stress values, which place them closer to the Perissodactyla, but also high average stress values, aligning them with the ruminants. For orthal biting configurations, *L*. *glama* plots close to the ruminants in all analyses. These results are consistent with the cluster analysis, which plots the Tylopoda together with the Perissodactyla. Considering the dominance of the phylogenetic signal as well as the specific digestive physiology, it is no surprise that both tylopods show specific traits, different from the Ruminantia. The lower stress values observed in the Tylopoda mandibles suggest greater morphological robusticity and may indicate greater reliance on comminution immediately following ingestion than in other ruminating taxa. Ruminatia meanwhile, appear to shift comminution to later rumination cycles, when cuds are soft and water soaked, thus requiring less chewing force. In fact, differences in the digestive-washing system between Ruminatia and Tylopoda could also explain the different biomechanical response of the mandible [86].

Further, our FEA data suggest that within the order Perissodactyla taxa also cluster according to dietary trait. This pattern is most notable amongst the Rhinocerotidae; *D*. *bicornis* and *D*. *sumatrensis* (general browsers) share a subcluster while *C*. *simum* and *E*. *quagga* (general grazers) also show close affinities. These results suggest that diet has influenced the biomechanical signal in Rhinocerotidae to a greater degree than any of the other ungulate families investigated in this study.

Interestingly, tapirs (*T*. *terrestris)* differ from the other perissodactyla. Cluster analysis suggests that *T*. *terrestris* has affinities with this group, possibly due to a strong phylogenetic signal [30], however it is almost consistently isolated in the lower left space of all intervals method biplots (but see Fig 8, Case 3), suggesting significant differences with the rest of the Perissodactyla. In particular, *T*. *terrestris* has the lowest average and peak stress values (see Fig 3 and S1 and S2 Tables of the Supplementary information). Moreover, the distribution of stress (Fig 2) shows more areas of low stress values, especially in the lateral bite configuration. Fig 8 sustains these results showing low values of average stress in tapir in both lateral and orthal bite. But, compared the other species in lateral occlusion, values are not too much lower (specifically compared with *D*. *sumatrensis*, *D*. *bicornis* or *C*. *dromedarius*) while in orthalbite values are the lowest by far. These results are due to a lack of she-cutting functionality in tapirs that need to be compensated with high orthal loads that require large masseters and masseter insertions areas. The high ortal loads result in significant attritional wear in tapirs that does not occur in the perissodactyls with a lateral power stroke like equids and rhinocerontids. These data confirms that the mandible of *T*. *terrestris* can be not particularly adapted to perform the lateral mode of power stroke and thus a more robust mandible should be necessary for comminution its cuts performing the orthal occlusion during chewing [87].Finally, average and peak stress values (see Fig 3 and S1 and S2 Tables of the Supplementary information) indicate that *Litocranius walleri* has the most fragile mandible. This finding is not surprising because *L*. *walleri*, a highly selective folivorous browser [88] forages on soft and easy to comminute. In browsing ruminants, compression of the bolus extraction of cell contents is accomplished by lateral occlusal movements which take advantage of the compression basins formed between enamel ridge 2 and 3 (e.g. [89]). The mandible (and brachydont cheek teeth) of *L*. *walleri* are hence assumed to not be adapted to withstand high bite forces or loads, and thus show the highest stress patterns when compared to mixed-feeding or grazing taxa among the ruminating group.

In summary, we conclude that the biomechanics of the ungulate mandible are influenced by both diet and phylogeny although more taxa might be needed to expand this conclusion. In general, a stronger phylogenetical signal seems to explain why Perissodactyla have stiffer mandibles than Artiodactyla. This difference is most pronounced between Perissodactyla and ruminant species. Perissodactyla rely heavily on thoroughly chewing their food upon ingestion, demanding the generation of high bite forces, while ruminants shift comminution to later rumination cycles when cuds are soft and water soaked, requiring lower bite forces to obtain physiologically sufficient mechanical disintegration. We therefore suggest that ruminants can afford to chew sloppily regardless of ingesta, while hindgut fermenters cannot.

Additionally, our data suggest a second adaptive layer resulting from diet suggesting that, within the orders Artiodactyla and Perissodactyla, mandibular morphologies also reflect the masticatory demands of specific ingesta (e.g. Typlopoda, Rhinocerotidae). A similar observation was made by [27], who found that mandible movement reflects a mammal’s phylogenetic history as well as its current feeding behaviour. Our findings thus agree with classical interpretations of ungulate mandibular morphology [6,17,27–29].

## Supporting information

S1 Table FEA results for the lateral biting cases. Number of mandible mesh elements and statistics: Arithmetic Mean (AM), Mesh-Weighted Arithmetic Mean (MWAM), Percentage Error of the Arithmetic Mean (PEofAM), Median (M), Mesh-Weighted Median (MWM), Percentage Error of the Median (PEofM) and the value quartiles (M25, M50, M75 and M95) according to Marcé-Nogué et al. 2016
(DOCX)

S2 Table FEA results for the orthal biting cases. Number of mandible mesh elements and statistics: Arithmetic Mean (AM), Mesh-Weighted Arithmetic Mean (MWAM), Percentage Error of the Arithmetic Mean (PEofAM), Median (M), Mesh-Weighted Median (MWM), Percentage Error of the Median (PEofM) and the value quartiles (M25, M50, M75 and M95) according to Marcé-Nogué et al. 2016
(DOCX)

S3 Table Convergence of the R2 values of the PC scores. Each value is the R2 for a different pair of PCAs of the correlation matrix. Each PC was correlated with the equivalent PC of the PCA developed using a larger number of intervals.
(DOCX)

S4 Table Percent of the variance of the PC scores. For the six biting cases
(DOCX)

S1 Document Intervals’ method data of Von Mises stress when N = 100 M for the six biting cases. (XLSX)

S1 Fig Plots displaying the first two PCs of the different PCAs for N = 10, 25, 50, 75 and 100 for case 1. Lateral biting in the first molar. The species are coloured by order: blue: Perissodactyla and brown Cetartiodactyla. The axes of each pair of PCs are in the same scale.
(TIF)

S2 Fig Plots displaying the first two PCs of the different PCAs for N = 10, 25, 50, 75 and 100 for case 2. Lateral biting in the second molar. The species are coloured by order: blue: Perissodactyla and brown Cetartiodactyla. The axes of each pair of PCs are in the same scale.
(TIF)

S3 Fig Plots displaying the first two PCs of the different PCAs for N = 10, 25, 50, 75 and 100 for case 3. Lateral biting in the third molar. The species are coloured by order: blue: Perissodactyla and brown Cetartiodactyla. The axes of each pair of PCs are in the same scale.
(TIF)

S4 Fig Plots displaying the first two PCs of the different PCAs for N = 10, 25, 50, 75 and 100 for case 4. Orthal biting in the first molar. The species are coloured by order: blue: Perissodactyla and brown Cetartiodactyla. The axes of each pair of PCs are in the same scale.
(TIF)

S5 Fig Plots displaying the first two PCs of the different PCAs for N = 10, 25, 50, 75 and 100 for case 5. Orthal biting in the second molar. The species are coloured by order: blue: Perissodactyla and brown Cetartiodactyla. The axes of each pair of PCs are in the same scale.
(TIF)

S6 Fig Plots displaying the first two PCs of the different PCAs for N = 10, 25, 50, 75 and 100 for case 6. Orthal biting in the third molar. The species are coloured by order: blue: Perissodactyla and brown Cetartiodactyla. The axes of each pair of PCs are in the same scale.
(TIF)
