# Supplementary material for: Why ruminating ungulates chew sloppily: Biomechanics discern a phylogenetic pattern
Source: PLoS One. 2019 Apr 17;14(4):e0214510. doi: 10.1371/journal.pone.0214510 (PMC6469769; doi:10.1371/journal.pone.0214510)
Supplement: S1 Table — Number of mandible mesh elements and statistics: Arithmetic Mean (AM), Mesh-Weighted Arithmetic Mean (MWAM), Percentage Error of the Arithmetic Mean (PEofAM), Median (M), Mesh-Weighted Median (MWM), Percentage Error of the Median (PEofM) and the value quartiles (M25, M50, M75 and M95) according to Marcé-Nogué et al. 2016 (DOCX) [file pone.0214510.s001.docx]

| **Case 1: Lateral biting in molar 1** | | | | | | | | | | | |
| --- | --- | --- | --- | --- | --- | --- | --- | --- | --- | --- | --- |
| **Specie** | **Jaw Elements** | **AM** | **MWAM** | **PEofAM** | **M** | **MWM** | **PEofM** | **M25** | **M50** | **M75** | **M95** |
| *A. buselaphus* | 597315 | 3.7934 | 3.7833 | 0.2668 | 2.8042 | 2.7261 | 2.8649 | 1.6424 | 2.8042 | 4.6783 | 10.9630 |
| *A. melampus* | 133087 | 4.0333 | 4.0057 | 0.6870 | 2.3817 | 2.3043 | 3.3599 | 1.3727 | 2.3817 | 4.6864 | 13.7740 |
| *C. dromedarius* | 477353 | 2.0926 | 2.0868 | 0.2758 | 1.6688 | 1.6395 | 1.7894 | 1.0319 | 1.6688 | 2.6308 | 5.8353 |
| *C. simum* | 562722 | 2.7709 | 2.7603 | 0.3862 | 2.2696 | 2.2151 | 2.4600 | 1.5040 | 2.2696 | 3.4225 | 6.4975 |
| *D. bicornis* | 345997 | 2.1623 | 2.1522 | 0.4659 | 1.8448 | 1.7956 | 2.7400 | 1.1939 | 1.8448 | 2.7116 | 4.8175 |
| *D. sumatrensis* | 331444 | 2.0421 | 2.0349 | 0.3533 | 1.7534 | 1.7034 | 2.9353 | 1.0619 | 1.7534 | 2.7016 | 4.7751 |
| *E. quagga* | 401415 | 2.4759 | 2.4619 | 0.5688 | 2.0273 | 1.9479 | 4.0781 | 1.0244 | 2.0273 | 3.3001 | 6.3307 |
| *G. camelopardalis* | 578161 | 3.5844 | 3.5843 | 0.0030 | 2.7311 | 2.6781 | 1.9788 | 1.4017 | 2.7311 | 4.9313 | 9.7606 |
| *L. glama* | 323990 | 2.4254 | 2.4172 | 0.3386 | 1.7992 | 1.7789 | 1.1356 | 1.1541 | 1.7992 | 3.0490 | 7.0306 |
| *L. walleri* | 280382 | 4.2577 | 4.2413 | 0.3885 | 3.0706 | 3.0082 | 2.0736 | 1.5932 | 3.0706 | 5.7733 | 11.5780 |
| *T. terrestris* | 423208 | 1.4305 | 1.4234 | 0.4978 | 1.1915 | 1.1757 | 1.3446 | 0.7494 | 1.1915 | 1.9280 | 3.2691 |
| **Case 2: Lateral biting in molar 2** | | | | | | | | | | | |
| *A. buselaphus* | 597315 | 3.9323 | 3.9196 | 0.3228 | 2.8384 | 2.7901 | 1.7311 | 1.4427 | 2.8384 | 5.2911 | 11.4560 |
| *A. melampus* | 133088 | 3.4386 | 3.4165 | 0.6447 | 2.2753 | 2.1702 | 4.8406 | 1.1791 | 2.2753 | 3.9940 | 11.4050 |
| *C. dromedarius* | 477344 | 1.7280 | 1.7234 | 0.2652 | 1.3754 | 1.3491 | 1.9512 | 0.8709 | 1.3754 | 2.1715 | 4.7612 |
| *C. simum* | 562753 | 2.3266 | 2.3180 | 0.3705 | 1.9261 | 1.8788 | 2.5198 | 1.2365 | 1.9261 | 2.8775 | 5.5145 |
| *D. bicornis* | 345997 | 1.8369 | 1.8290 | 0.4324 | 1.5870 | 1.5386 | 3.1479 | 0.9692 | 1.5870 | 2.3182 | 4.0929 |
| *D. sumatrensis* | 331444 | 1.7585 | 1.7526 | 0.3360 | 1.5197 | 1.4742 | 3.0864 | 0.8867 | 1.5197 | 2.3789 | 4.0914 |
| *E. quagga* | 401449 | 2.3372 | 2.3228 | 0.6180 | 1.9676 | 1.8910 | 4.0493 | 0.9807 | 1.9676 | 3.1152 | 5.8869 |
| *G. camelopardalis* | 578161 | 3.1400 | 3.1402 | 0.0094 | 2.3953 | 2.3354 | 2.5649 | 1.1745 | 2.3953 | 4.3893 | 8.5716 |
| *L. glama* | 323990 | 2.1111 | 2.1031 | 0.3830 | 1.5851 | 1.5669 | 1.1647 | 0.9559 | 1.5851 | 2.6992 | 6.1068 |
| *L. walleri* | 280382 | 3.9595 | 3.9430 | 0.4191 | 3.0512 | 2.9719 | 2.6673 | 1.5347 | 3.0512 | 5.3583 | 10.1190 |
| *T. terrestris* | 423208 | 1.2077 | 1.2018 | 0.4901 | 1.0089 | 0.9935 | 1.5460 | 0.5675 | 1.0089 | 1.6601 | 2.8708 |
| **Case 3: Lateral biting in molar 3** | | | | | | | | | | | |
| *A. buselaphus* | 597315 | 2.9194 | 2.9104 | 0.3090 | 2.2376 | 2.1715 | 3.0458 | 1.1532 | 2.2376 | 3.7172 | 8.6008 |
| *A. melampus* | 133087 | 2.8887 | 2.8710 | 0.6188 | 1.9627 | 1.8757 | 4.6356 | 1.0280 | 1.9627 | 3.4303 | 9.3223 |
| *C. dromedarius* | 477359 | 1.3501 | 1.3460 | 0.3041 | 1.0525 | 1.0360 | 1.5927 | 0.6308 | 1.0525 | 1.7120 | 3.7646 |
| *C. simum* | 562722 | 1.9457 | 1.9389 | 0.3502 | 1.6123 | 1.5736 | 2.4593 | 0.9843 | 1.6123 | 2.4114 | 4.7792 |
| *D. bicornis* | 345997 | 1.3780 | 1.3727 | 0.3874 | 1.1469 | 1.1196 | 2.4384 | 0.6600 | 1.1469 | 1.7673 | 3.2569 |
| *D. sumatrensis* | 331444 | 1.4312 | 1.4264 | 0.3388 | 1.2278 | 1.1978 | 2.5064 | 0.6894 | 1.2278 | 1.9749 | 3.3087 |
| *E. quagga* | 401415 | 2.2262 | 2.2113 | 0.6737 | 1.8607 | 1.7761 | 4.7622 | 0.8997 | 1.8607 | 2.9286 | 5.7321 |
| *G. camelopardalis* | 578161 | 2.6624 | 2.6624 | 0.0003 | 2.0628 | 2.0054 | 2.8623 | 0.9159 | 2.0628 | 3.7271 | 7.3719 |
| *L. glama* | 323990 | 1.8050 | 1.7969 | 0.4512 | 1.2985 | 1.2718 | 2.0955 | 0.7078 | 1.2985 | 2.3223 | 5.2755 |
| *L. walleri* | 280382 | 3.5233 | 3.5063 | 0.4848 | 2.7817 | 2.7046 | 2.8520 | 1.3973 | 2.7817 | 4.6304 | 8.7415 |
| *T. terrestris* | 423208 | 1.0076 | 1.0029 | 0.4736 | 0.8125 | 0.8017 | 1.3361 | 0.4273 | 0.8125 | 1.3649 | 2.5961 |
