# Supplementary material for: Why ruminating ungulates chew sloppily: Biomechanics discern a phylogenetic pattern
Source: PLoS One. 2019 Apr 17;14(4):e0214510. doi: 10.1371/journal.pone.0214510 (PMC6469769; doi:10.1371/journal.pone.0214510)
Supplement: S2 Table — Number of mandible mesh elements and statistics: Arithmetic Mean (AM), Mesh-Weighted Arithmetic Mean (MWAM), Percentage Error of the Arithmetic Mean (PEofAM), Median (M), Mesh-Weighted Median (MWM), Percentage Error of the Median (PEofM) and the value quartiles (M25, M50, M75 and M95) according to Marcé-Nogué et al. 2016 (DOCX) [file pone.0214510.s002.docx]

| **Case 4: Orthal biting in molar 1** | | | | | | | | | | | |
| --- | --- | --- | --- | --- | --- | --- | --- | --- | --- | --- | --- |
| **Specie** | **Jaw Elements** | **AM** | **MWAM** | **PEofAM** | **M** | **MWM** | **PEofM** | **M25** | **M50** | **M75** | **M95** |
| *A. buselaphus* | 597315 | 5.4889 | 5.4681 | 0.3817 | 3.0794 | 3.0299 | 1.6337 | 1.2541 | 3.0794 | 7.5859 | 18.6858 |
| *A. melampus* | 133089 | 4.5997 | 4.5652 | 0.7551 | 2.5100 | 2.4044 | 4.3931 | 1.0734 | 2.5100 | 6.0208 | 16.4931 |
| *C. dromedarius* | 477353 | 5.1561 | 5.1403 | 0.3073 | 4.5597 | 4.4615 | 2.2011 | 2.6975 | 4.5597 | 6.7363 | 12.0180 |
| *C. simum* | 562713 | 4.4879 | 4.4736 | 0.3214 | 3.1775 | 3.1403 | 1.1846 | 1.9628 | 3.1775 | 5.6572 | 12.7970 |
| *D. bicornis* | 345997 | 3.8739 | 3.8659 | 0.2085 | 2.8249 | 2.7288 | 3.5217 | 1.3119 | 2.8249 | 5.4554 | 10.7740 |
| *D. sumatrensis* | 331445 | 4.2569 | 4.2343 | 0.5317 | 3.3098 | 3.2423 | 2.0805 | 1.5323 | 3.3098 | 6.0572 | 11.1660 |
| *E. quagga* | 401416 | 4.0540 | 4.0337 | 0.5043 | 2.8297 | 2.7309 | 3.6176 | 0.8128 | 2.8297 | 5.7745 | 12.3650 |
| *G. camelopardalis* | 578161 | 4.3230 | 4.3100 | 0.3016 | 2.8874 | 2.8376 | 1.7547 | 0.8281 | 2.8874 | 6.1831 | 13.8830 |
| *L. glama* | 323990 | 5.0220 | 5.0136 | 0.1671 | 3.2342 | 3.1520 | 2.6054 | 1.5716 | 3.2342 | 6.7531 | 15.7030 |
| *L. walleri* | 280382 | 6.8138 | 6.7508 | 0.9331 | 4.1709 | 4.0824 | 2.1677 | 2.0789 | 4.1709 | 7.8741 | 22.6460 |
| *T. terrestris* | 423208 | 2.7365 | 2.7292 | 0.2670 | 2.2371 | 2.1762 | 2.7989 | 1.0569 | 2.2371 | 3.8373 | 7.1057 |
| **Case 5: Orthal biting in molar 2** | | | | | | | | | | | |
| *A. buselaphus* | 597315 | 4.8180 | 4.7982 | 0.4133 | 2.5739 | 2.5204 | 2.1232 | 1.0035 | 2.5739 | 6.5552 | 16.9760 |
| *A. melampus* | 133088 | 3.9505 | 3.9165 | 0.8674 | 1.8384 | 1.7453 | 5.3339 | 0.7642 | 1.8384 | 5.0616 | 15.1330 |
| *C. dromedarius* | 477353 | 4.4417 | 4.4267 | 0.3391 | 3.7322 | 3.6565 | 2.0703 | 2.0130 | 3.7322 | 5.9920 | 11.1320 |
| *C. simum* | 562708 | 4.0004 | 3.9872 | 0.3306 | 2.7699 | 2.7330 | 1.3502 | 1.6234 | 2.7699 | 5.0203 | 11.7890 |
| *D. bicornis* | 345997 | 3.4676 | 3.4568 | 0.3139 | 2.2150 | 2.1790 | 1.6510 | 1.1369 | 2.2150 | 4.8750 | 10.2830 |
| *D. sumatrensis* | 331444 | 3.7984 | 3.7762 | 0.5860 | 2.6687 | 2.6525 | 0.6114 | 1.2971 | 2.6687 | 5.4823 | 10.3240 |
| *E. quagga* | 401415 | 3.9695 | 3.9484 | 0.5330 | 2.7093 | 2.6265 | 3.1525 | 0.8207 | 2.7093 | 5.6823 | 12.1390 |
| *G. camelopardalis* | 578161 | 3.8833 | 3.8727 | 0.2735 | 2.2874 | 2.2454 | 1.8705 | 0.5438 | 2.2874 | 5.6832 | 13.1890 |
| *L. glama* | 323990 | 4.2493 | 4.2373 | 0.2851 | 2.5293 | 2.4476 | 3.3341 | 0.9858 | 2.5293 | 5.7441 | 14.3550 |
| *L. walleri* | 280382 | 6.3525 | 6.2863 | 1.0525 | 3.5711 | 3.5206 | 1.4346 | 1.7221 | 3.5711 | 7.4495 | 21.9958 |
| *T. terrestris* | 423208 | 2.4562 | 2.4502 | 0.2453 | 1.8162 | 1.7786 | 2.1140 | 0.8171 | 1.8162 | 3.5094 | 6.8903 |
| **Case 6: Orthal biting in molar 3** | | | | | | | | | | | |
| *A. buselaphus* | 597315 | 7.1205 | 7.0992 | 0.3004 | 5.1434 | 5.0669 | 1.5091 | 3.1023 | 5.1434 | 8.8614 | 20.2300 |
| *A. melampus* | 133088 | 3.3148 | 3.2805 | 1.0442 | 1.1089 | 1.1144 | 0.4956 | 0.5313 | 1.1089 | 4.1810 | 13.5021 |
| *C. dromedarius* | 477353 | 3.4179 | 3.4041 | 0.4066 | 2.4617 | 2.4061 | 2.3108 | 1.1196 | 2.4617 | 4.7405 | 9.9807 |
| *C. simum* | 562766 | 3.7161 | 3.7046 | 0.3112 | 2.5139 | 2.4868 | 1.0889 | 1.4800 | 2.5139 | 4.5497 | 11.1000 |
| *D. bicornis* | 345997 | 3.1628 | 3.1492 | 0.4300 | 2.0284 | 2.0209 | 0.3709 | 1.2276 | 2.0284 | 3.9648 | 9.8880 |
| *D. sumatrensis* | 331444 | 3.4457 | 3.4230 | 0.6637 | 2.2912 | 2.2612 | 1.3267 | 1.2168 | 2.2912 | 4.8657 | 9.6185 |
| *E. quagga* | 401415 | 3.9610 | 3.9383 | 0.5751 | 2.5455 | 2.4917 | 2.1592 | 0.9563 | 2.5455 | 5.6813 | 12.1590 |
| *G. camelopardalis* | 578057 | 3.3099 | 3.2985 | 0.3467 | 1.6952 | 1.6691 | 1.5637 | 0.3312 | 1.6952 | 4.6769 | 12.0560 |
| *L. glama* | 323990 | 3.4637 | 3.4508 | 0.3748 | 1.5876 | 1.5449 | 2.7625 | 0.4643 | 1.5876 | 4.7716 | 13.0590 |
| *L. walleri* | 280382 | 5.8150 | 5.7469 | 1.1855 | 2.8698 | 2.8331 | 1.2970 | 1.2948 | 2.8698 | 6.8902 | 21.4354 |
| *T. terrestris* | 423208 | 2.1887 | 2.1830 | 0.2588 | 1.4346 | 1.4188 | 1.1136 | 0.7338 | 1.4346 | 2.9158 | 6.7001 |
