# Supplementary material for: Why ruminating ungulates chew sloppily: Biomechanics discern a phylogenetic pattern
Source: PLoS One. 2019 Apr 17;14(4):e0214510. doi: 10.1371/journal.pone.0214510 (PMC6469769; doi:10.1371/journal.pone.0214510)
Supplement: S3 Table — Each value is the R2 for a different pair of PCAs of the correlation matrix. Each PC was correlated with the equivalent PC of the PCA developed using a larger number of intervals. (DOCX) [file pone.0214510.s003.docx]

| **Case 1: Lateral biting in molar 1** | **PC1** | **PC2** |
| --- | --- | --- |
| PCA 10 vs. PCA 15 | 0.9986 | 0.9928 |
| PCA 15 vs. PCA 25 | 0.9995 | 0.9967 |
| PCA 25 vs. PCA 50 | 0.9993 | 0.9930 |
| PCA 50 vs. PCA 75 | 0.9999 | 0.9992 |
| PCA 75 vs. PCA 100 | 0.9999 | 0.9996 |
| **Case 2: Lateral biting in molar 2** | **PC1** | **PC2** |
| PCA 10 vs. PCA 15 | 0.9989 | 0.9561 |
| PCA 15 vs. PCA 25 | 0.9994 | 0.9846 |
| PCA 25 vs. PCA 50 | 0.9995 | 0.9948 |
| PCA 50 vs. PCA 75 | 0.9999 | 0.9955 |
| PCA 75 vs. PCA 100 | 0.9999 | 0.9995 |
| **Case 3: Lateral biting in molar 3** | **PC1** | **PC2** |
| PCA 10 vs. PCA 15 | 0.9958 | 0.4619 |
| PCA 15 vs. PCA 25 | 0.9995 | 0.6418 |
| PCA 25 vs. PCA 50 | 0.9997 | 0.0159 |
| PCA 50 vs. PCA 75 | 0.9999 | 0.8630 |
| PCA 75 vs. PCA 100 | 0.9999 | 0.9898 |
| **Case 4: Orthal biting in molar 1** | **PC1** | **PC2** |
| PCA 10 vs. PCA 15 | 0.9968 | 0.9788 |
| PCA 15 vs. PCA 25 | 0.9988 | 0.9789 |
| PCA 25 vs. PCA 50 | 0.9995 | 0.9948 |
| PCA 50 vs. PCA 75 | 0.9999 | 0.9997 |
| PCA 75 vs. PCA 100 | 0.9999 | 0.9999 |
| **Case 5: Orthal biting in molar 2** | **PC1** | **PC2** |
| PCA 10 vs. PCA 15 | 0.9943 | 0.9836 |
| PCA 15 vs. PCA 25 | 0.9982 | 0.9591 |
| PCA 25 vs. PCA 50 | 0.9996 | 0.9923 |
| PCA 50 vs. PCA 75 | 0.9999 | 0.9998 |
| PCA 75 vs. PCA 100 | 0.9999 | 0.9999 |
| **Case 6: Orthal biting in molar 3** | **PC1** | **PC2** |
| PCA 10 vs. PCA 15 | 0.9896 | 0.9731 |
| PCA 15 vs. PCA 25 | 0.9935 | 0.9920 |
| PCA 25 vs. PCA 50 | 0.9994 | 0.9835 |
| PCA 50 vs. PCA 75 | 0.9999 | 0.9990 |
| PCA 75 vs. PCA 100 | 0.9999 | 0.9999 |
