# Supplementary material for: Why ruminating ungulates chew sloppily: Biomechanics discern a phylogenetic pattern
Source: PLoS One. 2019 Apr 17;14(4):e0214510. doi: 10.1371/journal.pone.0214510 (PMC6469769; doi:10.1371/journal.pone.0214510)
Supplement: S4 Table — For the six biting cases (DOCX) [file pone.0214510.s004.docx]

| **PC** | **% Variance** | | | | | |
| --- | --- | --- | --- | --- | --- | --- |
|  | **Case 1** | **Case 2** | **Case 3** | **Case 4** | **Case 5** | **Case 6** |
| 1 | 75.81 | 78.42 | 78.86 | 59.41 | 61.88 | 62.97 |
| 2 | 13.50 | 10.49 | 8.01 | 22.96 | 20.52 | 18.93 |
| 3 | 5.63 | 6.03 | 7.23 | 8.14 | 8.31 | 8.46 |
| 4 | 3.04 | 2.36 | 2.58 | 4.86 | 4.58 | 4.88 |
| 5 | 0.97 | 1.86 | 1.39 | 1.91 | 1.80 | 2.13 |
| **6** | 0.44 | 0.32 | 1.26 | 1.37 | 1.20 | 0.95 |
| 7 | 0.27 | 0.23 | 0.42 | 0.62 | 0.71 | 0.85 |
| 8 | 0.18 | 0.16 | 0.12 | 0.32 | 0.57 | 0.40 |
| 9 | 0.14 | 0.08 | 0.09 | 0.25 | 0.25 | 0.26 |
| 10 | 0.03 | 0.06 | 0.04 | 0.14 | 0.18 | 0.18 |
