# Supplementary figures and images for: Why ruminating ungulates chew sloppily: Biomechanics discern a phylogenetic pattern
Source: PLoS One. 2019 Apr 17;14(4):e0214510. doi: 10.1371/journal.pone.0214510 (PMC6469769; doi:10.1371/journal.pone.0214510)

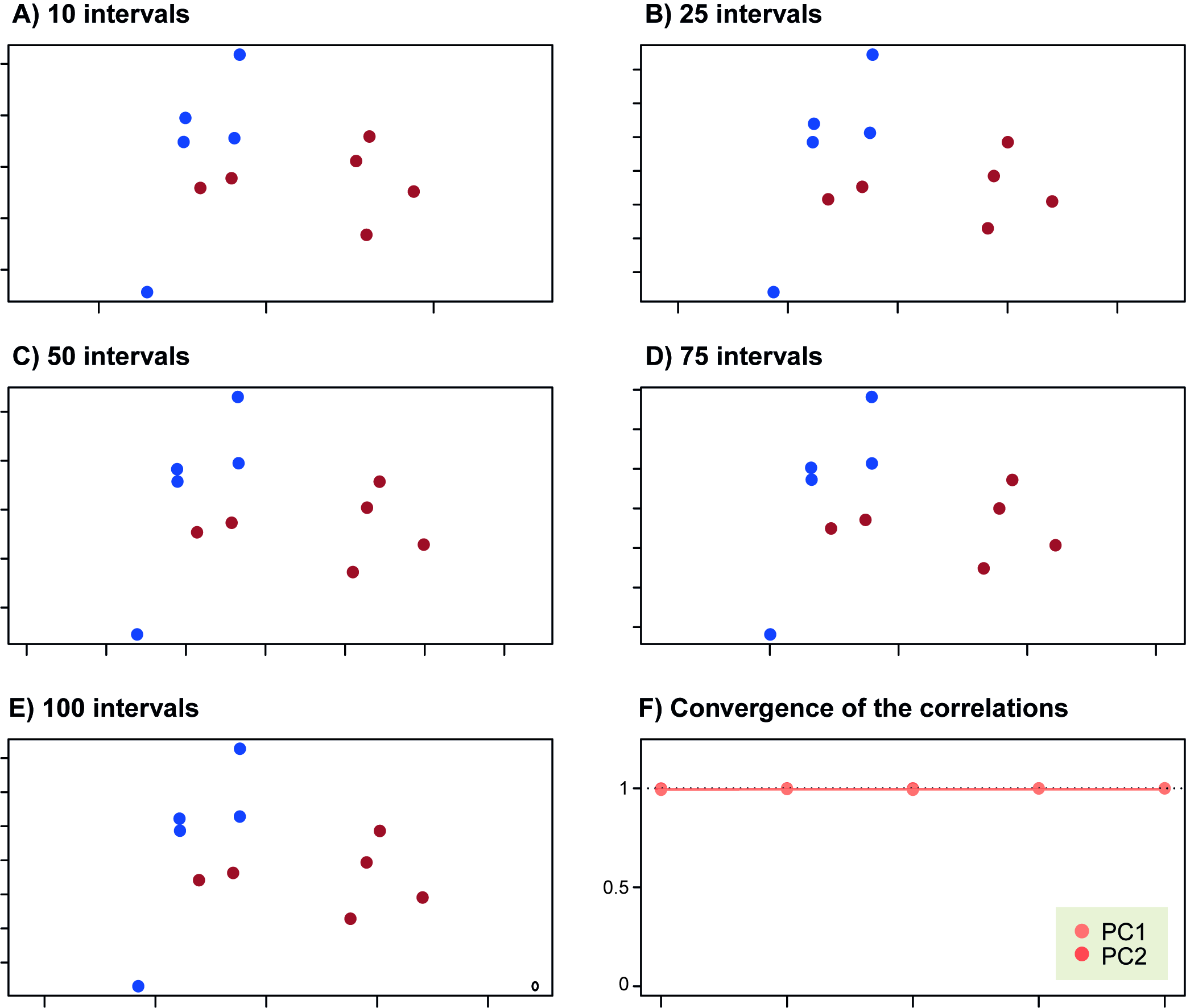

Supplement: S1 Fig — Lateral biting in the first molar. The species are coloured by order: blue: Perissodactyla and brown Cetartiodactyla. The axes of each pair of PCs are in the same scale. (TIF) [file pone.0214510.s006.tif]

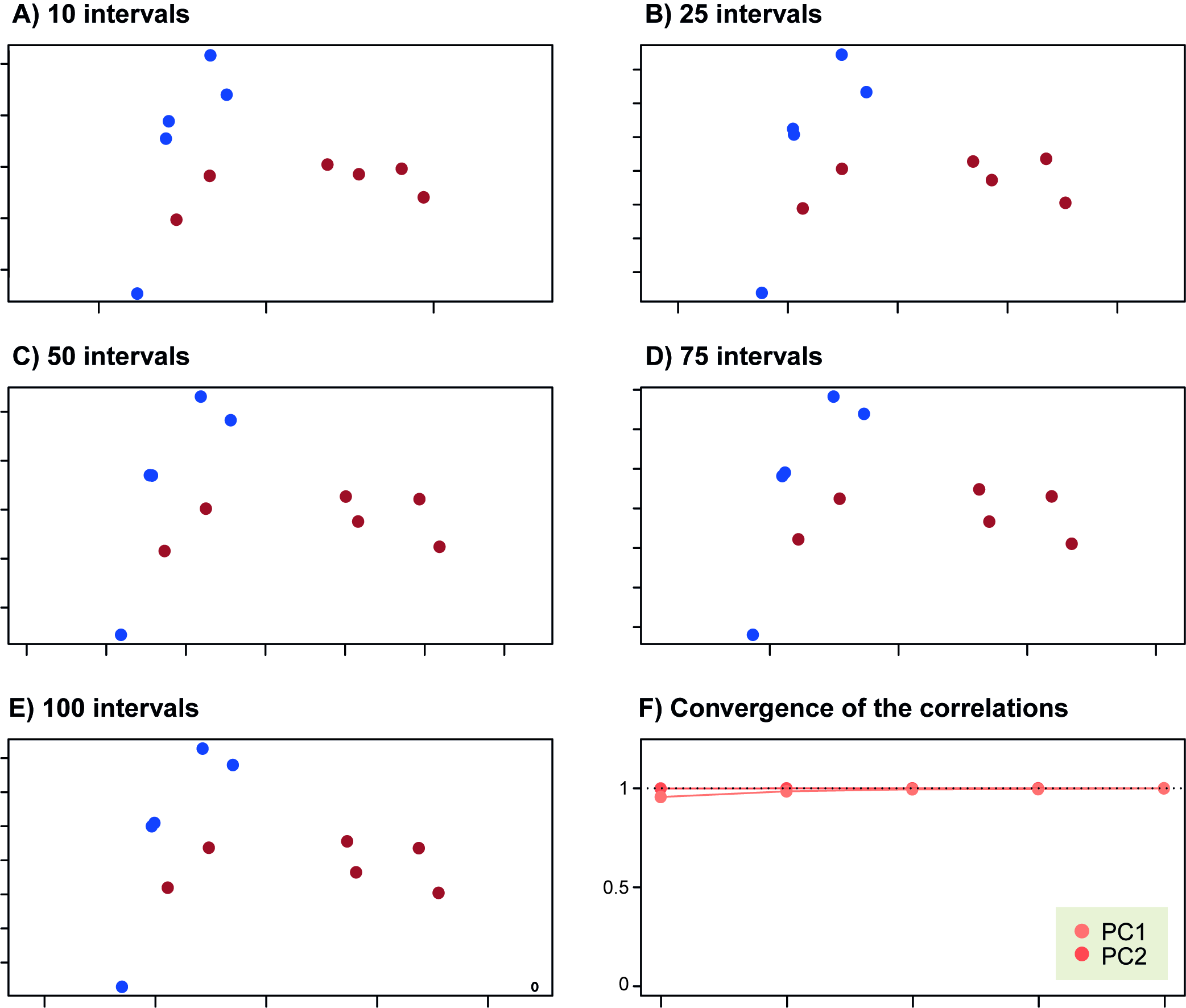

Supplement: S2 Fig — Lateral biting in the second molar. The species are coloured by order: blue: Perissodactyla and brown Cetartiodactyla. The axes of each pair of PCs are in the same scale. (TIF) [file pone.0214510.s007.tif]

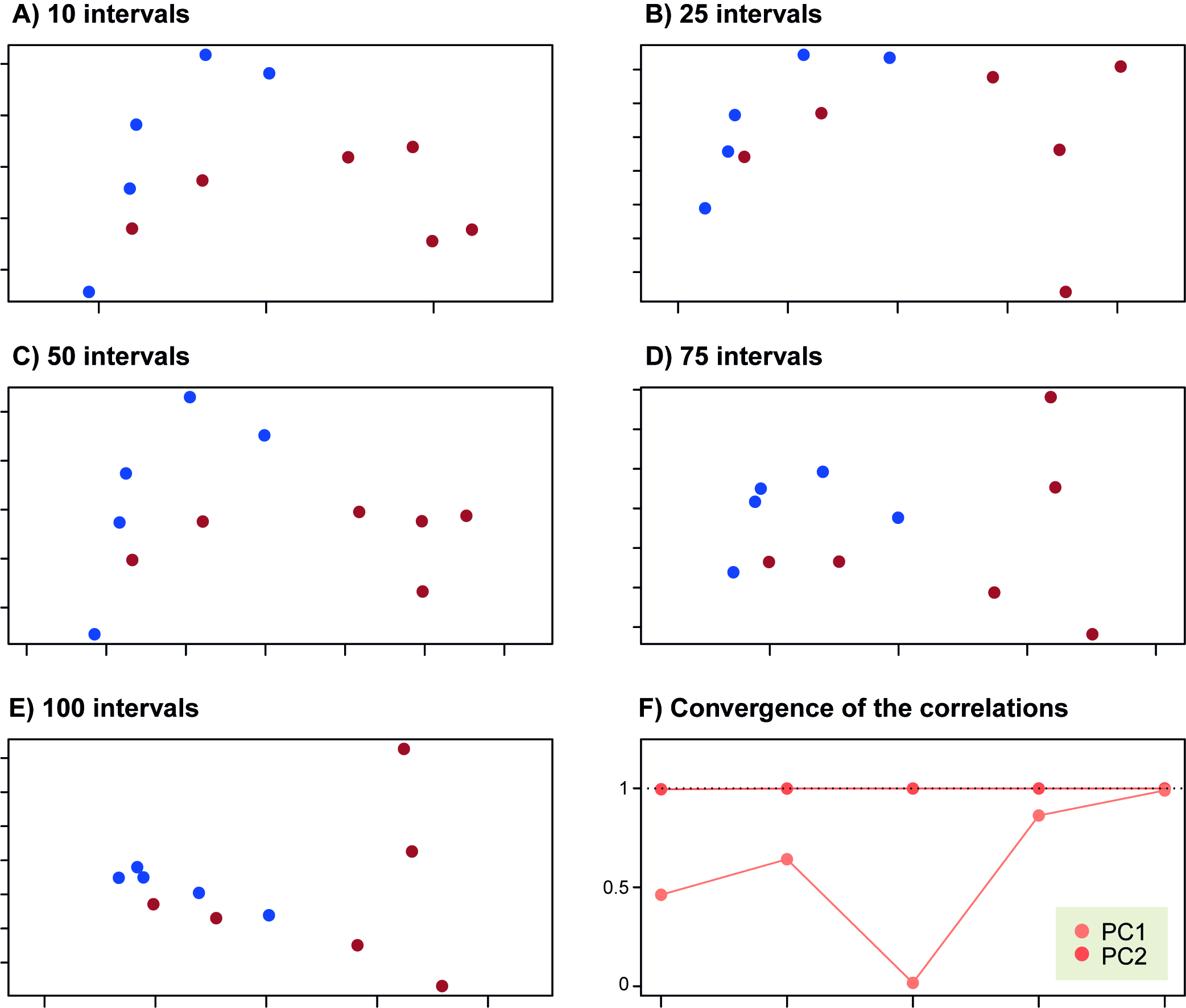

Supplement: S3 Fig — Lateral biting in the third molar. The species are coloured by order: blue: Perissodactyla and brown Cetartiodactyla. The axes of each pair of PCs are in the same scale. (TIF) [file pone.0214510.s008.tif]

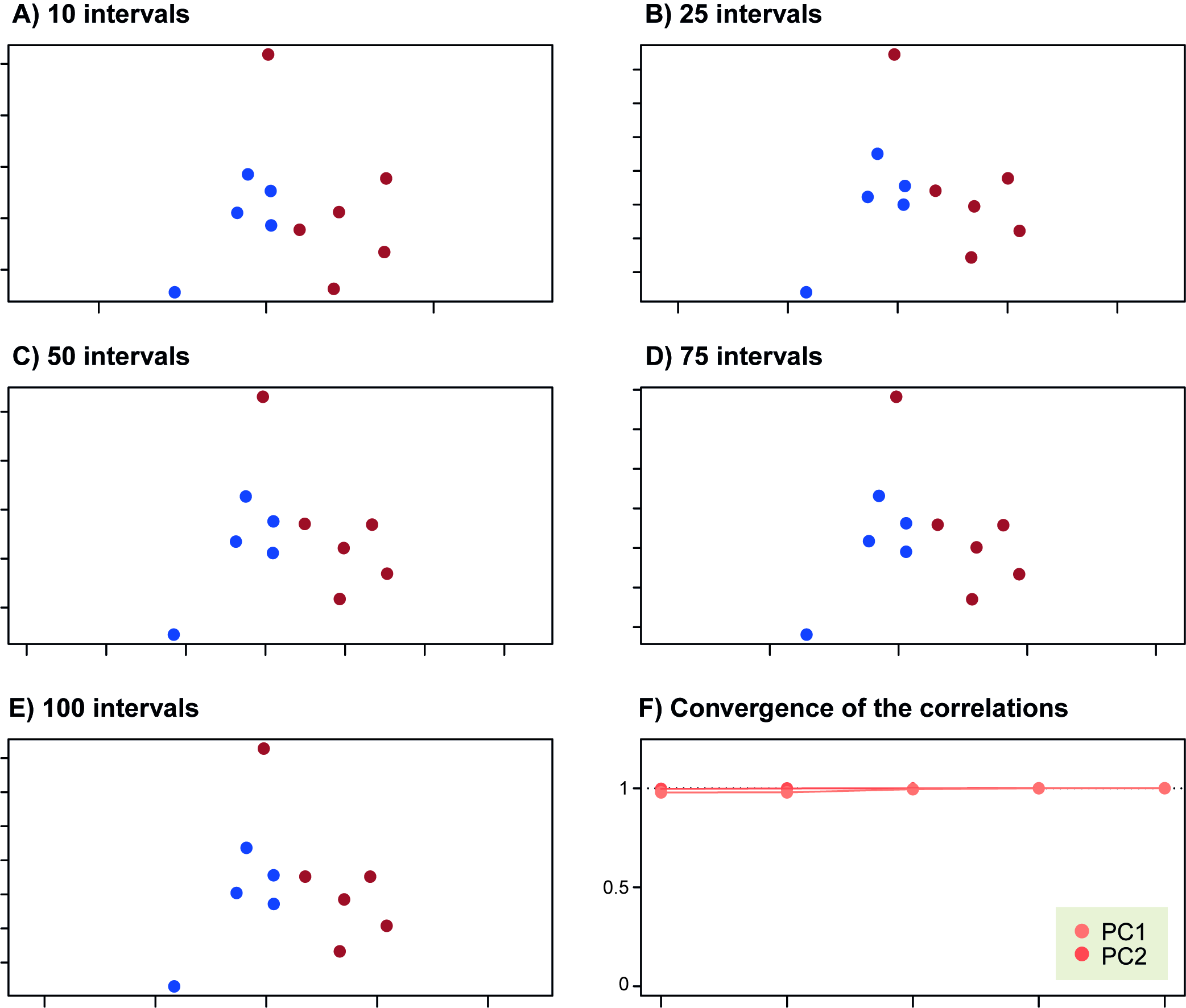

Supplement: S4 Fig — Orthal biting in the first molar. The species are coloured by order: blue: Perissodactyla and brown Cetartiodactyla. The axes of each pair of PCs are in the same scale. (TIF) [file pone.0214510.s009.tif]

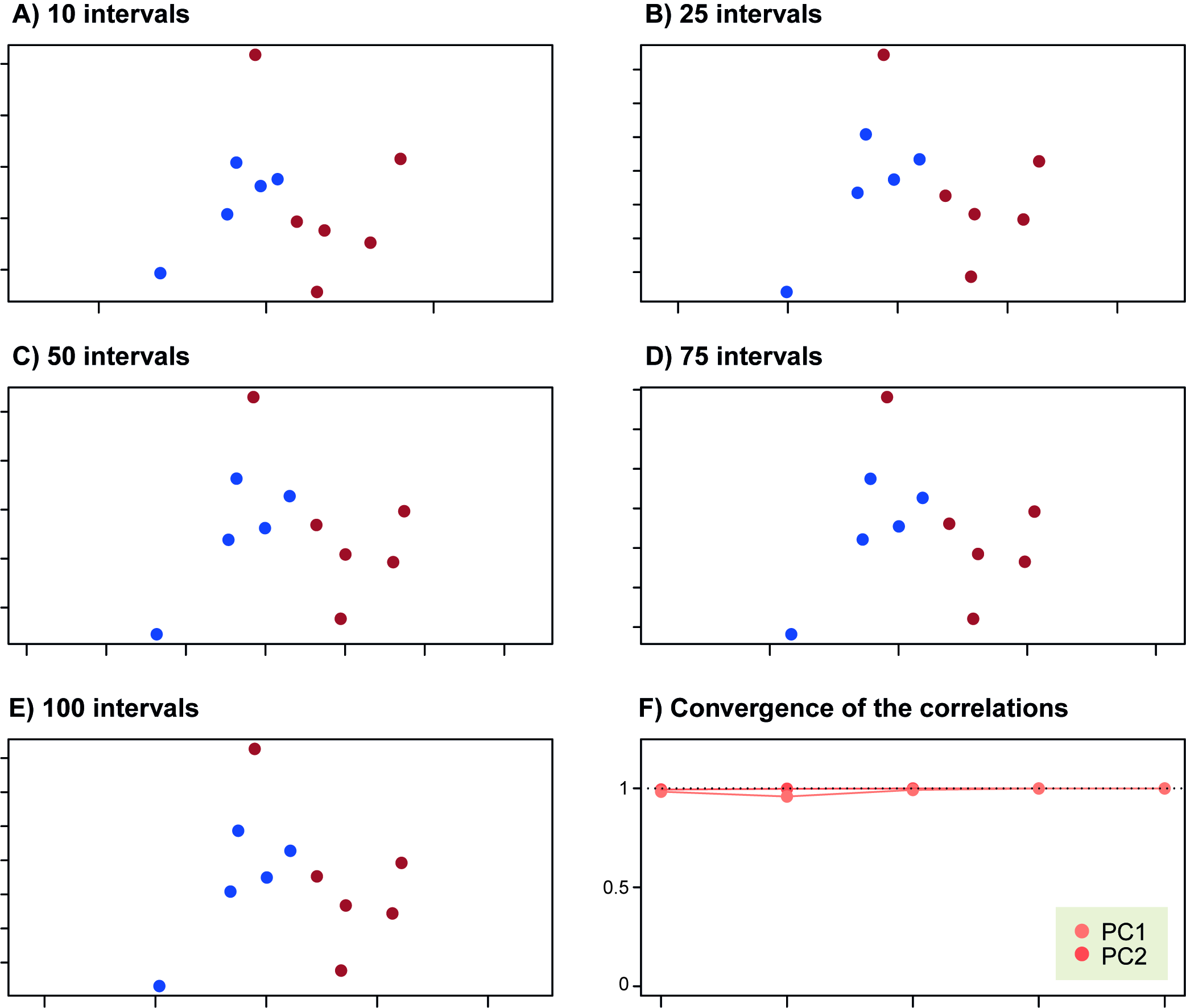

Supplement: S5 Fig — Orthal biting in the second molar. The species are coloured by order: blue: Perissodactyla and brown Cetartiodactyla. The axes of each pair of PCs are in the same scale. (TIF) [file pone.0214510.s010.tif]

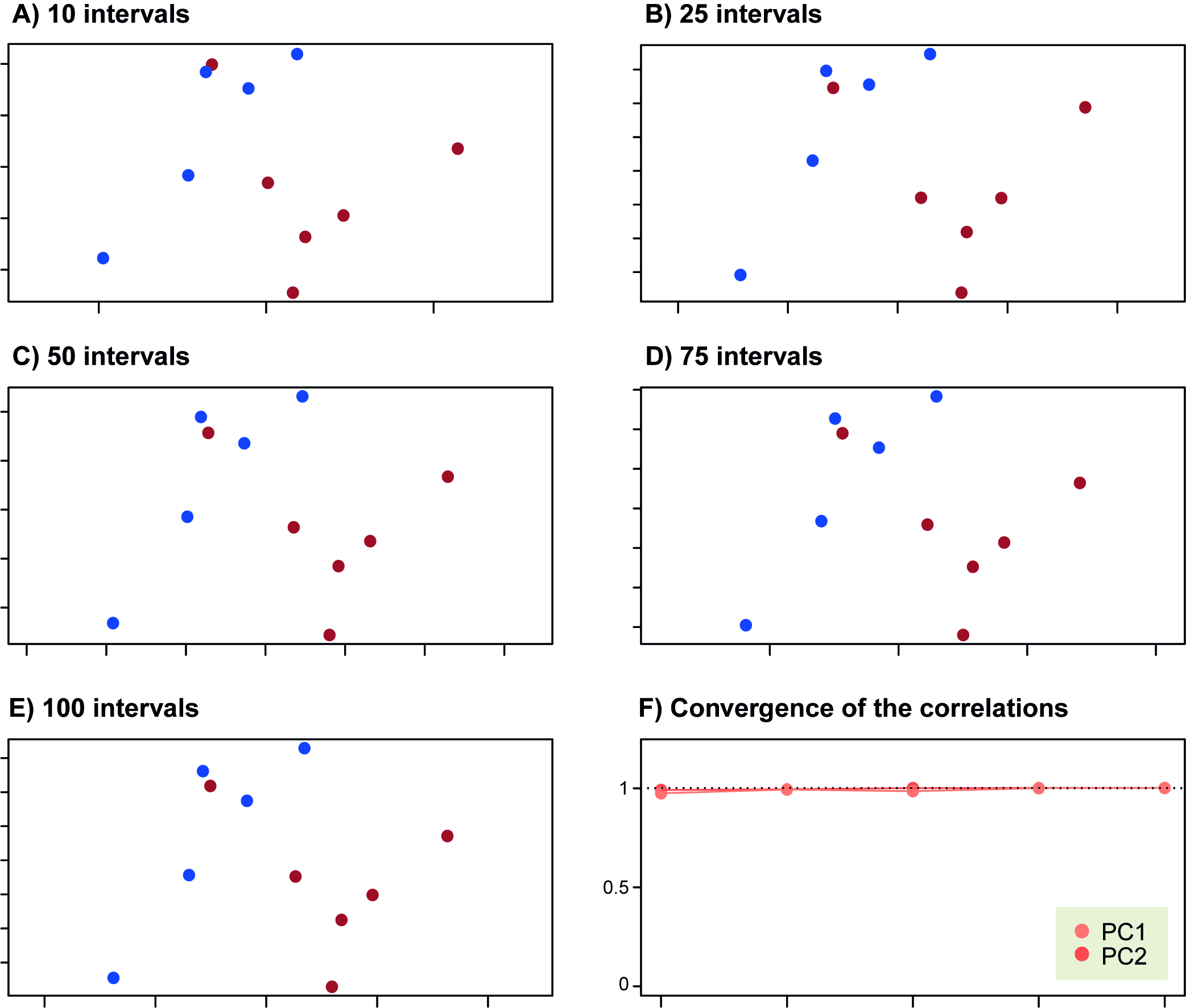

Supplement: S6 Fig — Orthal biting in the third molar. The species are coloured by order: blue: Perissodactyla and brown Cetartiodactyla. The axes of each pair of PCs are in the same scale. (TIF) [file pone.0214510.s011.tif]
